# A-RAF Kinase Functions in ARF6 Regulated Endocytic Membrane Traffic

**DOI:** 10.1371/journal.pone.0004647

**Published:** 2009-02-27

**Authors:** Elena Nekhoroshkova, Stefan Albert, Matthias Becker, Ulf R. Rapp

**Affiliations:** Institut für Medizinische Strahlenkunde und Zellforschung (MSZ), University of Würzburg, Würzburg, Germany; University of Geneva, Switzerland

## Abstract

**Background:**

*RAF* kinases direct ERK MAPK signaling to distinct subcellular compartments in response to growth factor stimulation.

**Methodology/Principal Findings:**

Of the three mammalian isoforms A-RAF is special in that one of its two lipid binding domains mediates a unique pattern of membrane localization. Specific membrane binding is retained by an N-terminal fragment (AR149) that corresponds to a naturally occurring splice variant termed DA-RAF2. AR149 colocalizes with ARF6 on tubular endosomes and has a dominant negative effect on endocytic trafficking. Moreover actin polymerization of yeast and mammalian cells is abolished. AR149/DA-RAF2 does not affect the internalization step of endocytosis, but trafficking to the recycling compartment.

**Conclusions/Significance:**

A-RAF induced ERK activation is required for this step by activating ARF6, as A-RAF depletion or inhibition of the A-RAF controlled MEK-ERK cascade blocks recycling. These data led to a new model for A-RAF function in endocytic trafficking.

## Introduction

RAF protein kinases were originally identified as viral oncogenes [1], [2] found in murine and avian retroviruses. *RAF* genes encode protein serine/threonine kinases [3], [4] that mediate transduction of extracellular mitogenic signals from activated Ras GTPases at the plasma membrane to a MAP kinase module (RAF-MEK-ERK), the mitogenic cascade (reviewed in [5]). As a result, complex physiological responses to growth factor stimulation take place at multiple cellular levels.

Insect genomes contain only a single *RAF* gene whereas vertebrates have refined RAF signaling and employ three isoforms that target ERK signalling to different subcellular compartments [6]. Specialized functions are reflected in differential regulation of RAF kinase activity [7] and varying phenotypes of RAF knockout mice [8]–[10]. The three RAF isoforms and their splice variants share common structural features comprising three conserved regions, CR1, CR2, CR3. The N-terminal CR1 encompasses the Ras binding domain (RBD) and the cysteine-rich domain (CRD), CR2 contains a conserved 14-3-3 binding motif and the C-terminally located CR3 encodes the kinase domain [11].

Whereas B- and C-RAF were studied extensively, little is known about A-RAF function. A-RAF is marked by a low basal kinase activity, which has been attributed to substitutions in its N-region, where tyrosine 296 plays a central role [12]. In contrast to animals with a genomic deletion of B- or C-RAF, A-RAF ^−/−^ mice are viable, but die perinatally, depending on the genetic background [8].

Budding yeast *Saccharomyces cerevisiae* is an established eukaryotic model organism that has played a key role in the elucidation of the MAP kinase signaling pathway. Despite the presence of two redundant RAS genes and at least 6 MAPK cascades [13], no RAF kinases are present in *S. cerevisiae*. Nevertheless, yeast was important for defining RAF function as an activator of a prototypical MAP kinase cascade in experiments that involved ectopic expression of C-RAF [14]. RAF isoforms are known to function as homo and heterodimers in mammalian cells [6], complicating assignment of individual function. Therefore yeast is an attractive system for investigation of a single RAF isoform.

Endocytosis is a process essential for many aspects of cellular life, including receptor internalization and recycling. ARF6 GTPase was shown to regulate endocytosis at several levels [15]. The activation state of ARF6 is determined by the bound nucleotide, GTP or GDP, which also affects intracellular localization. Nucleotide loading is regulated by specialized guanine nucleotide exchange factors (GEFs) and GTPase activating proteins (GAPs) that catalyze hydrolysis of bound GTP [16]. Endocytosis and signal transduction are known to be functionally linked and regulating each other (Von Zastrow and Sorkin, 2007, Polo and Di Fiore, 2006). ARF6 as a central regulator of endocytic trafficking was shown to activate ERK [17], [18]. Based on changes in endocytosis upon inhibition of ERK signaling Robertson et al. (2006) suggested a role of ERK signaling in the regulation of clathrin-independent endocytosis.

Here we describe the role of A-RAF in membrane trafficking and identify its function at a specific step of endocytosis. This work led to the discovery of a C-terminally truncated version of A-RAF, AR149 that strongly interfered with cell growth and polarization in yeast and with endocytosis and actin polymerization in mammalian cells. As this work was in progress two splicing isoforms of A-RAF, termed DA-RAF1,2 were described that act as natural inhibitors of RAS-ERK signaling during myogenic differentiation [19]. DA-RAF2 contains the first 153 aar of A-RAF and thus is nearly identical with AR149. AR149 localized specifically to the recycling endosomal compartments as confirmed by colocalization and coprecipitation with ARF6. Expression of AR149 interferes with recycling of endocytosed transferrin (Tfn) and with actin polymerization. siRNA-mediated depletion of endogenous A-RAF or inhibition of MEK by U0126 mimic AR149 function, supporting a role of A-RAF regulated ERK signaling at endosomes that is controlled by AR149/DA-RAF2 and targets ARF6.

## Materials and Methods

If not otherwise stated reagents were of p.a. purity (Sigma, USA), restriction enzymes were from New England Biolabs (USA) and Fermentas (Lithuania).

### Plasmids used in this study

Deletion mutants of A-RAF were generated by insertion of PCR products into the pUG36 plasmid. In the case of N-terminal mutants of A-RAF, BamHI and HindIII recognition sequences have been attached to the sequence of forward and reverse primers respectively. Primers for C-terminal mutants of A-RAF contained SmaI and HindIII respectively. Doubly truncated mutants contained BamHI and XhoI restriction sites. PCR products were generated using primers listed in Table S1.

For expression of the GFP fusion proteins in mammalian cells we used pEGFP-C-1 and pDS-Red2 (Invitrogen, USA). ARF6 constructs tagged with hemagglutinin (HA) in pLNCX, FLAG-EFA6 and GST-GGA3 were generous gift from Margaret Chou, University of Pennsylvania. GFP-tagged ARF6 was kindly provided by Antoine Galmiche. GFP-ARF6(Q67L) and GFP-ARF6(T27N) were generated by site directed mutagenesis (QuikChange, Stratagene) with the primers listed in Table S1. GFP-A-RAF in pEGFP-C1 was kindly donated by Angela Baljuls.

### Cell culture and cell fractionation

Media and reagents were purchased from Invitrogen (USA). HeLa, NIH 3T3 and COS7 cells were maintained in DMEM supplemented with 10% fetal bovine serum (FBS), penicillin and streptomycin. For starvation, DMEM was supplemented with 0,03% FBS. Cell transfections utilized jetPEI (Biomol).

Subcellular fractionation of HeLa cell lysates employed “ProteoExtract Subcellular Proteome Extraction Kit “(Calbiochem) according to manufacturer's instructions.

### Fluorescence Microscopy

Fluorescence microscopy was done with an Openlab software (Improvision, UK) controlled inverted DMIRBE microscope (Leica, Germany) with Leica oil immersion objective. All images were captured and stored as Openlab LIF files. Images were subsequently processed using Photoshop software.

#### Yeast live cell imaging

Yeast cells were transferred into a self-made chamber slide for imaging.

#### Fixed yeast cells imaging

Cells were fixed in 3.7% paraformaldehyde in PBS, washed and subsequently digested for 1 hour with lyticase (Sigma). After washing and mounting, samples were either stored at 4°C or processed for imaging.

#### Mammalian cells imaging

Cells were grown on cover slips, treated with growth factors or serum as indicated and subsequently fixed in 3.7% paraformaldehyde, permeabilized with 0.1% Triton X-100. Stainings were performed with specific antibodies and fluorescently labeled secondary antibodies.

### Indirect immunofluorescence after cytosol depletion

HeLa cells were grown on coverslips overnight. After two washes with PBS cells were treated with 0,05% digitonin in isotonic sucrose buffer for 4 min on ice [20]. After digitonin treatment, cells were fixed with 3.7% paraformaldehyde in microtubule-stabilizing buffer (MSB; 0.1 M PIPES, pH 6.9, 2 mM MgCl_2_, 2 mM EGTA), washed and subsequently permeabilized with 0,1% w/v Saponin in MSB with 0.5% BSA for 10 min. To stain non-cytosolic A-RAF, cells were incubated with anti-A-RAF antibodies (Santa Cruz, USA) in combination with anti β-Tubulin antibodies (Chemicon International) at concentration of 20 µg/ml in MSB buffer with 0,5% BSA and 0,1% Saponin at room temperature for 2 h. Unbound antibodies were removed by 3 washes with the same buffer. The coverslips were incubated with appropriate secondary antibody (conjugated to TRITC or CY5) diluted 1∶200 for 1 h. After three washes with MSB and brief wash with deionized water the coverslips were mounted using MOWIOL (Calbiochem, USA).

### siRNA-mediated depletion of human A-RAF

For generation of A-RAF specific siRNA we used “X-tremeGENE siRNA Dicer Kit” (Roche). Prepared siRNA mix containied about 15 different siRNAs. The target sequence for human A-RAF located at the 3′end of A-RAF coding region was from “Human esiRNA resource” (German Resource Center for Genome). Scrambled siRNA was from QIAGEN. Transfection was carried out using 2 µg of siRNA mixture and 10 µl of “X-tremeGENE siRNA Transfection Reagent” (Roche) for 6-well culture plates, according to the instructions provided by the manufacturer.

### Transferrin internalization

HeLa cells were grown on coverslips overnight, transfected with either GFP or GFP-fused AR149, ARF6(Q67L), ARF6(T27N) respectively for 48 h.

The cells were pre-incubated in serum-free medium for 1 h at 37°C. For continuous Tfn uptake, the cells were incubated in internalization medium (HBSS medium plus 1% BSA) containing 5 µg/ml Alexa Fluor 546-conjugated human Tfn (Invitrogen) at 37°C for indicated time. After Tfn internalization the cells were extensively washed three times with ice-cold PBS and fixed with 3.7% PFA.

### Yeast strains and techniques

Protease deficient strains cI3 ABYS 86, BJ 5459 (Yeast Genetic Stock Center, University of California, Berkeley) were used in order to prevent degradation of expressed proteins.

Standard protocols for yeast growth, transformation and manipulations were employed [21]. Yeast transformation was performed by a modified litihum acetate method. The following plasmids were used in this study: pUG36 and pUG36 for detection of GFP-fusion proteins; pEG-KT for galactose-inducible expression of GST-fusion proteins. Membrane fractionation on 20–35% sucrose gradients and indirect immunofluorescence microscopy were done as described previously [22].

### Immunoprecipitation of GFP-AR149 and ARF6

COS7 cells were transfected with GFP-AR149 and HA-ARF6wt, HA-ARF6(Q67L) or HA-ARF6(T27N) respectively with jetPEI (Biomol). After 24 h, cells were lysed in ARF6 lysis buffer (50 mM Tris-HCl pH 7.0, 2 mM MgCl_2_, 100 mM NaCl, 10% Glycerol, 0.75% NP-40) containing protease inhibitors. To avoid high signal from heavy and light chains of antibodies on the Western blot we used Mouse IgG TruhBlot Set (NatuTec) including beads and secondary HRP-conjugated antibody. The clarified lysates were divided into two equilibrated parts, each of them was incubated for 2 h at 4°C with anti-GFP or anti-HA antibody respectively (Santa Cruz Biotechnology, USA). Next, the probes were precipitated with mouse-TruhBlot agarose (NatuTec) for 1 h at 4°C. Beads were washed three times in the lysis buffer with 0.2% NP-40 and protease inhibitors.

### GGA3 pulldown assay

Activated ARF6•GTP was monitored by binding to its effector GGA3 as described previously [23]. Briefly, COS7 cells were co-transfected with HA-ARF6 plus indicated plasmids using jetPEI and grown for 24 h. EGF stimulation was performed after 24 h starvation in 0.03% serum with 100 ng/ml EGF (Cell System Biotechnologie Vertrieb) for 10 min at 37°C. Clarified lysates were incubated with 25 µg of GST-GGA3 immobilized on glutathione-Sepharose beads for 2 h at 4°C. The beads were washed three times with PBS, resuspended in SDS PAGE loading buffer and boiled. Bound proteins were size-fractionated by SDS-PAGE and detected by immunoblotting.

## Results

### Isotype specific distribution of RAF proteins in yeast

**Figure 1:**
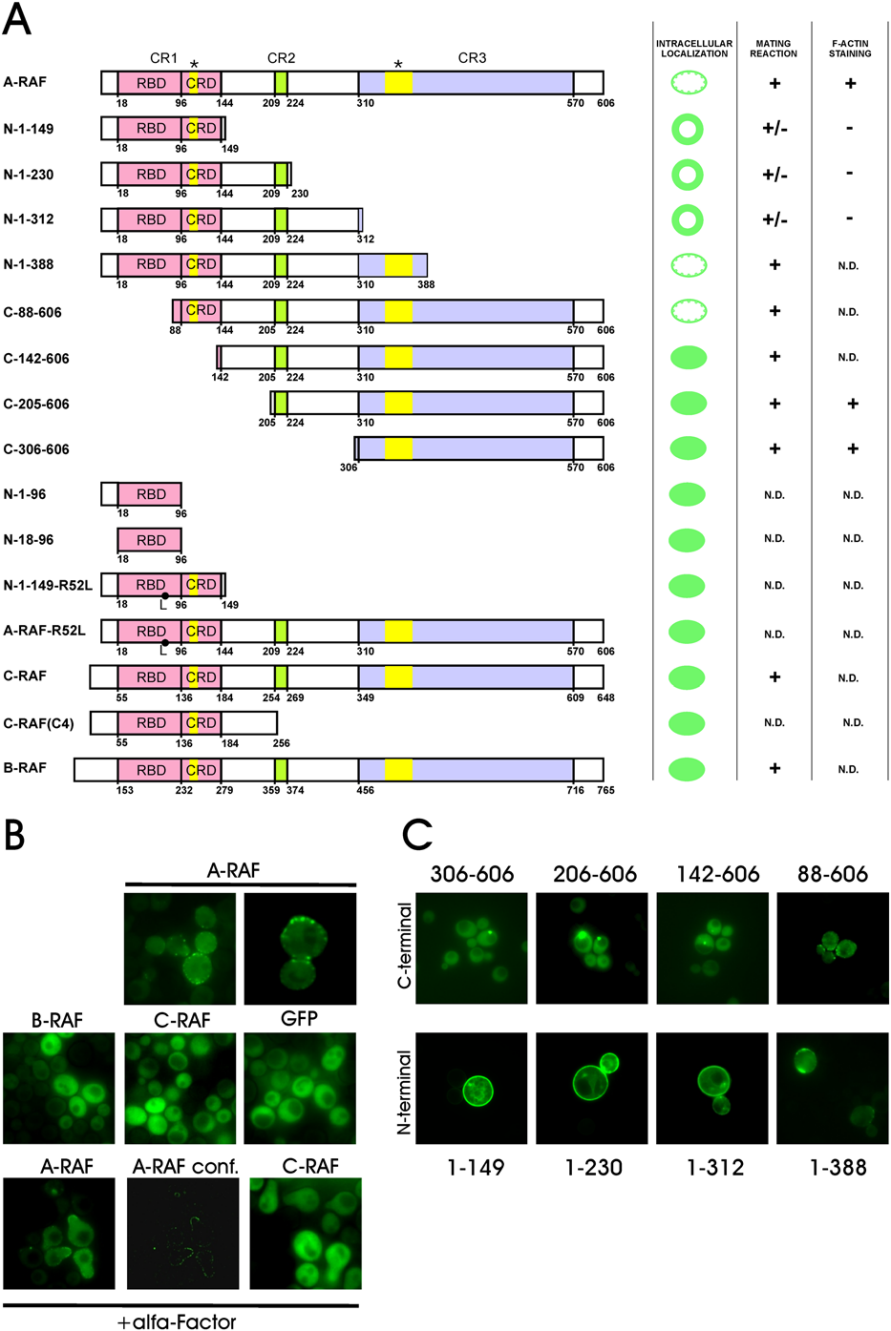
Differential localization of wild type and mutant forms of RAF in yeast. A. N- and C-terminal deletions or point mutations were expressed as GFP fusions in yeast. Staining patterns of mutants are shown on the right. The largest C- and N-terminal deletions showing the “wild type” localisation are 1–388 and 88–606. Lipid binding domains (see also Figure S2), are indicated by yellow boxes. B. Localization of RAF isoforms in yeast. Only GFP-A-RAF localizes to small dots in the cell cortex, which accumulate to tips of small buds and to necks of larger buds (upper row). Induced polarization of yeast by α-factor leads to relocation of GFP-A-RAF to the tip of mating projections called shmoos (lowermost row). C. Localization patterns of C- and N-terminal deletion mutants. The smallest N-terminal deletion (88–606) retains wild type distribution. C-terminal deletions that lost the presumptive PA binding motif, while retaining the PtdIns(4,5)P_2_ binding motif in CRD (see the scheme above) are homogenously distributed to plasma membrane. The same magnification was used throughout.

All three human *RAF* genes were fused with the C-terminus of green fluorescent protein (GFP) and expressed under the control of a moderately inducible *MET25* promoter in *S. cerevisiae*. GFP-B-RAF and GFP-C-RAF fluorescence was evenly distributed in the cytosol of induced cells. In contrast, A-RAF fluorescence localized to distinct punctate cortical structures, which were polarized toward the tips of small buds and bud necks of larger buds (Figure 1B). Treatment with α-factor, concentrated GFP-A-RAF at the tips of mating projections termed shmoos (Figure 1B). Sucrose density gradient centrifugation was employed to test the observed differences in localization of GFP-RAF proteins. Consistent with the flourescence data, both, C- and -B-RAF segregated with cytosolic proteins in a sucrose density gradient, whereas A-RAF was predominantly found in the heavy membrane fraction (Figure S1A). Taken together, of the three RAF isoforms only A-RAF interacts with yeast membrane components or proteins that are polarized during cell division and mating.

### Multiple lipid binding motifs of A-RAF are required for its unique localization

To test, which A-RAF domain was responsibe for its subcellular distribution in yeast we generated a set of amino- (N-) and carboxy-terminal (C-) A-RAF deletion mutants fused to GFP. The localization of each construct was controlled by fluorescence microscopy. A schematic overview of their subcellular distribution is presented in Figure 1A, right panel. Representative micrographs are shown in Figure 1C. Expression and correct size of the deletion mutants was ascertained by Western blot analysis (Figure S1B).

Three types of staining patterns were observed: (i) homogeneous cytosolic as seen with full length B- and C-RAF (see 142–606 construct in Figure 1C), (ii) a punctate pattern (see 88–606 construct in Figure 1C), and (iii) an intermediate pattern, with intensive homogeneous labeling of plasma membranes (see Figure 1C, 1–149 construct as an example).

Two different lipid-binding domains have previously been identified in the structure of C-RAF. A phosphatidic acid (PA) binding domain, located in the N-terminal part of CR3 [24], [25], and a phosphatidyl serine (PS) binding domain located in the cysteine-rich region of CR1 [25], [26]. Moreover, deletion of CR1 of A-RAF (which encompasses RBD+CRD) led to loss of A-RAF-specific binding to PtdIns(4,5)P_2_ suggesting a third lipid binding site [27]. As these sites are well conserved among RAF isoforms, we mutagenized their key residues, which have previously been shown to affect the interaction of C-RAF with PA and PS (Figure S2) [28]. Substitution of basic residues in the PS site led to localization of mutant protein in the cytosol. This pattern strongly resembled that of N-terminally truncated deletion mutants. In contrast, mutant protein with substitution of basic residues in the PA binding site led to homogeneous distribution in the plasma membrane, the typical pattern for C-terminally truncated mutants (Figure S2). We conclude that the A-RAF specific punctate pattern requires both, the PS/PIP_2_ and PA lipid binding sites.

### Expression of AR149 in yeast causes growth inhibition, defects in cell polarization, in nuclear morphology and in membrane trafficking

**Figure 2:**
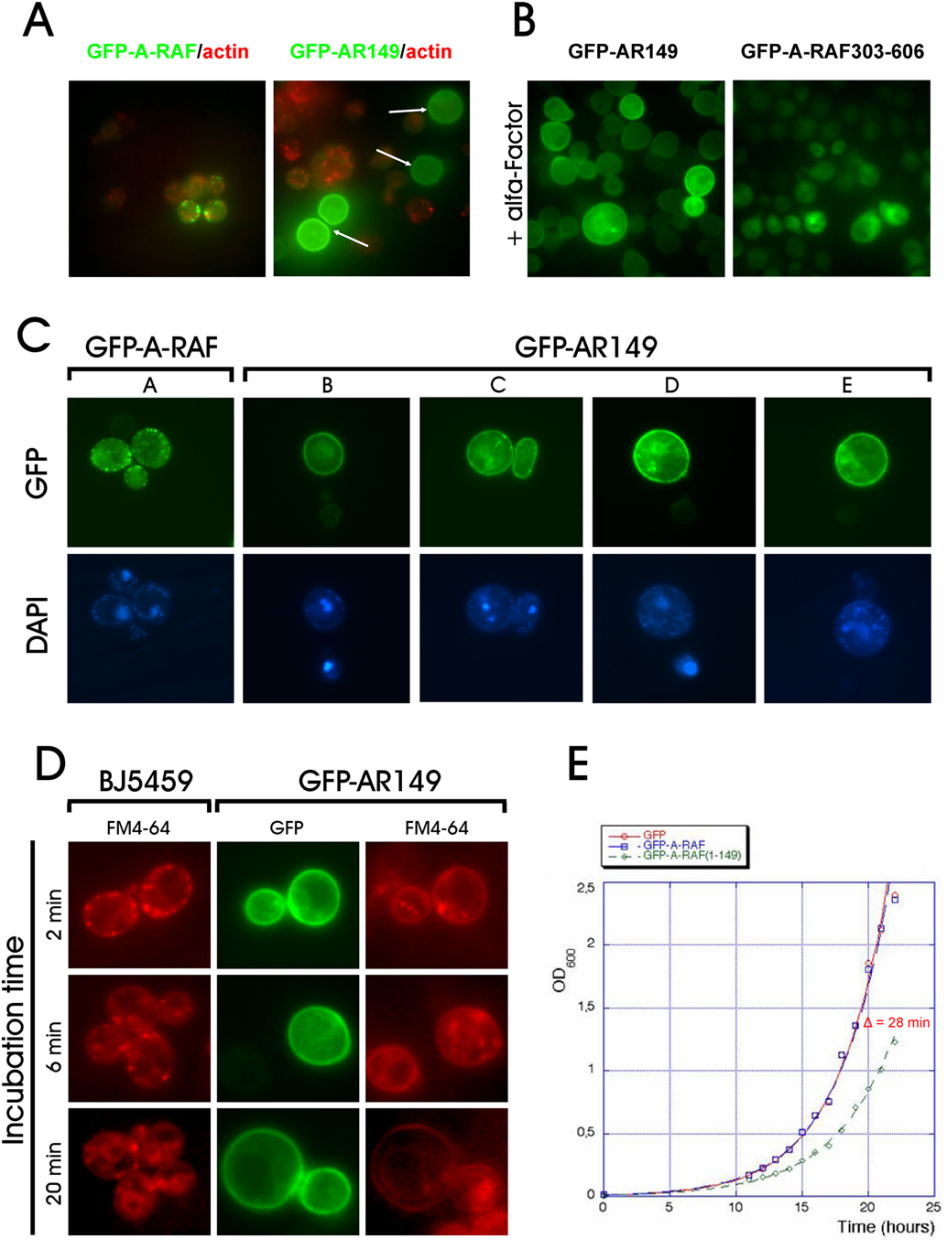
Physiological effects of AR149 overexpression in yeast. A. Cells expressing high amounts of GFP-AR149 fail to form cortical actin patches. Cells expressing GFP-A-RAF or GFP-AR149 were grown, fixed as described in “Experimental procedures”. Polymerized actin was visualized with Alexa Fluor 546-conjugated Phalloidin. Note that cells expressing higher levels of GFP-AR149 but not of full length A-RAF (bright green cells marked with arrows) lost polymerized actin. B. GFP-AR149 expression inhibits ∝-factor induced shmoo formation. Cells transformed with pUG36-AR149 (right) or A-RAF(303–606) (left) were treated with ∝-factor for 2 h. Note increased overall size and absence of shmoos in cells expressing GFP-AR149. C. Changes in nuclear morphology of GFP-AR149 expressing cells. DNA of the nucleus and mitochondria was stained with DAPI. Morphology of nuclei of AR149 expressors varied from fragmented (left) to completely dispersed (right). D. FM-4-64 uptake is affected in AR149-expressing yeast. Yeast transformed with pUG36-AR149 and untransformed control were incubated with lipophilic styryl dye FM 4–64 for indicated time, washed and observed by fluorescence microscopy. Transition of the red fluorescence from periplasmic endocytic sites to vacuoles is clearly visible at 20 minutes in control cells, but not in GFP-AR149 expressing cells. E. Growth inhibition of AR149 expressing cells. Cells transformed with pUG36, pUG36-A-RAF or pUG36-AR149 were grown under selective conditions in fresh selective medium. Cell proliferation was monitored by spectrophotometry.

In the course of deletion analysis, we noted that cells expressing several C-terminally truncated mutants showed homogenous membrane localization, different from that of full-length protein. In addition, a significant fraction of cells expressing the C-terminal deletion mutants was larger and apolar (round in shape instead of oval) as compared to GFP or GFP-A-RAF expressing cells (see Figure 2A, B). Polarization during cell growth by directed membrane trafficking is essential for cell division, therefore we hypothesized that GFP-AR149 expressing cells may exhibit retarded cell cycle progression and as a consequence loss of viability. To test this, we grew GFP, GFP-A-RAF and GFP-AR149 in liquid culture, under promoter de-repressing conditions. Cells expressing GFP-AR149 showed sustained growth inhibition. The difference of 28 minutes in duplication time confirms that expression of GFP-AR149, but not that of GFP or full-length A-RAF exhibits an inhibitory effect on expressing cells (Figure 2E). We stained actin and DNA in GFP-AR149 and GFP-A-RAF expressing cells. In agreement with the apolar phenotype, no polymerized actin could be detected in the large round cells expressing higher levels of GFP-AR149 (Figure 2A arrows). Intriguingly, in the same cells (large, apolar) no intact nuclei could be detected by DAPI staining (Figure 2C). Viability tests showed about 20% decrease in survival rate of GFP-A-RAF149 expressing cells, which is in good agreement with the percentage of apolar cells found in the culture. In a FM4-64 uptake assay we found that yeasts expressing the AR-149 were affected in endocytosis (Figure 2D) conversely endocytosis was unaffected when GFP-A-RAF was expressed (Figure S1D).

To test the effect of the fusion partner and of high level expression of A-RAF and of AR149 on yeasts, these proteins were expressed as GST-fusions under the strongly inducible GAL10 promoter. Cells were transformed with GST-A-RAF or GST-AR149, streaked on glucose- (“promoter off”) or galactose (“promoter on”) media and grown at 30°C. Figure S1C shows that whereas all three strains grew well in the repressive medium, in the inductive medium the GST-AR149 expressing strain did not grow.

Taken together, AR149, but not full-length A-RAF, localizes homogeneously to the plasma membrane, and blocks yeast cell polarization, actin polymerization, and endocytosis, resulting in cell growth inhibition. Moreover cells overexpressing AR149 from a strong GAL10 promoter were not viable (Figure S1C).

### Localization of A-RAF and AR149 in human cell lines

The dramatic differences in localization between B- and C-RAF versus A-RAF and its N-terminal fragment, AR149, observed in yeast, prompted us to analyze the latter proteins in mammalian cell lines. AR149 expression had a strong negative effect on actin polymerization in yeast (Figure 2A). To test this effect in mammalian cells, we chose NIH 3T3 cell line known for extended fibers of polymerized actin called stress fibers. Similar to yeast, stress fibers in NIH 3T3 cells were diminished by GFP-AR149 expression (Figure S3). This effect was also described for DA-RAF1, 2 by Yokoyama et al. (2007).

**Figure 3:**
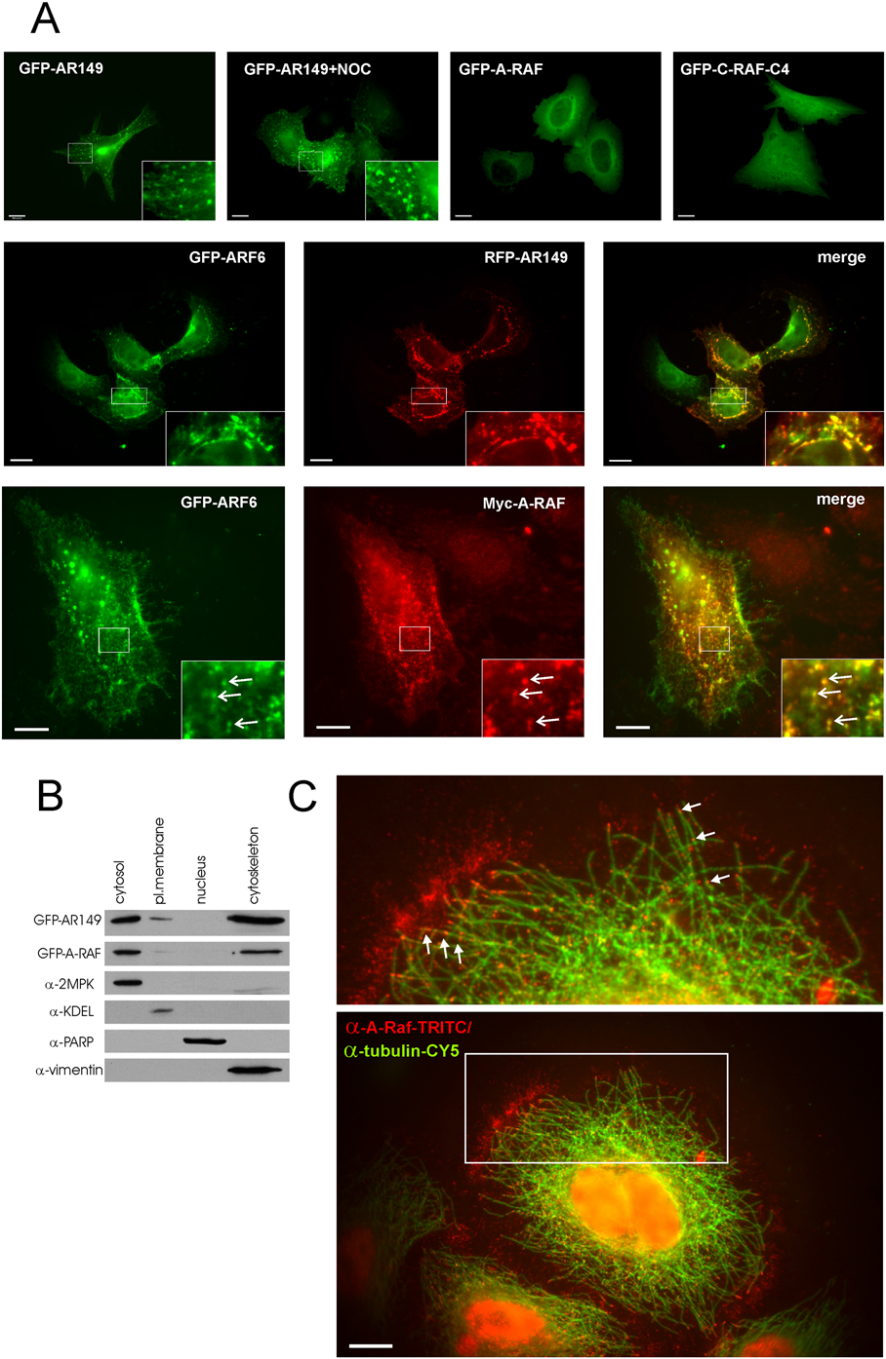
Localization of GFP-A-RAF, GFP-AR149 and endogenous A-RAF in mammalian cells. A. Top row: HeLa cells were transiently transfected with pEGFP bearing the indicated genes for 2 days. GFP fusion proteins were detected by fluorescence microscopy. GFP-A-RAF is present throughout the cytoplasm and accumulates around the nucleus. In contrast, GFP-AR149 labels punctate structures often aligned on strings. Strings were disassembled by treatment with Nocodazole. A C-RAF fragment orthologous to AR149 is GFP-C-RAF(C4). Representative images are shown. Scale bar = 10 µm. Middle row: HeLa cells were cotransfected with RFP-AR149 and GFP-ARF6 as described above. RFP and GFP fluorescences were recorded separately. Bottom row: HeLa cells were cotransfected with Myc-A-RAF and GFP-ARF6. After two days transfected cells were treated with digitonin to extract cytosol and processed for detection of A-RAF and ARF6 as described in Figure 3C. Boxed areas are shown at higher magnification. Arrows indicate vesicles with colocalization. Representative images are shown. Scale bar = 10 µm. B. Cells were fractionated using “ProteoExtract Subcellular Proteome Extraction Kit” (Calbiochem) and analyzed by immunoblotting. antibodies against following proteins were used as compartmental markers: vimentin as cytoskeletal marker, PARP as nuclear marker, 2MPK as cytosolic marker. Both A-RAF and AR149 are co-fractionating predominantly with cytoskeleton and cytosol. Small portion of AR149 was also found in plasma membrane fraction. C. HeLa cells were treated with digitonin to extract cytosol. After fixation and washing, immunofluorescence microscopy with antibodies against A-RAF and against β-tubulin was carried out. The boxed area is shown at higher magnification. Small A-RAF positive vesicles are at the periphery and line microtubules (arrows). Representative images are shown. Scale bar = 10 µm.

We next examined the subcellular localization of A-RAF and AR149 in HeLa cells. Cells were transfected with GFP-tagged A-RAF and AR149 respectively. For control, an N-terminal fragment of C-RAF (aar 1–256) described originally as a dominant inhibitory RAF mutant [29] that corresponds to AR149 (named C-RAF-C4) was included in the analysis. As observed in yeast, AR149 was unique in that it labeled the plasma membrane. Additionally in HeLa cells “beads on a string” structures (Figure 3A, top panel) were seen, demonstrating isotype specificity of the lipid binding site in CR1 as B- and C-RAF were reported to localize instead to ER/Golgi complex or mitochondria respectively [30], [20]. Disruption of microtubules by Nocodazole treatment separated beads from the strings (Figure 3A, top panel). The stained vesicular tubular structures most likely represent recycling endosomes. To test this hypothesis, we studied the localization of RFP-AR149 and that of an established endosomal marker, ARF6. As documented in Figure 3A, middle panel, there is a high degree of colocalization between RFP-AR149 and GFP-ARF6. ARF6 was reported to regulate central steps of endocytosis and recycling of endocytosed material by early and recycling endosomes (see [16] for review). Like nearly all GTPases, ARF6 functions as a molecular switch alternating between GTP-bound (active) and GDP bound (inactive) states. Consequently, overexpressed GTP-, GDP-locked or nucleotide-free mutants of ARF6 (Q67L, T27N and N121I respectively) have distinct dominant effects on endosomal morphology and endocytosis [31].

In co-transfected cells we found colocalization of RFP-AR149 (Figure 3A, middle panel) or wild type A-RAF (Figure 3A, bottom panel) with GFP-ARF6 in tubular vesicular structures.

In summary, AR149 and wild type A-RAF, but not C-RAF-C4 localizes to tubular endosomes, as proven by specific colocalization with ARF6.

### A fraction of endogenous A-RAF localizes to vesicles located along microtubuli

Considering the dominant negative effect of AR149 on endocytosis in yeast, we set out to determine whether endogenous A-RAF in HeLa cells was localized on endocytic vesicles.

After cytosol depletion, fixation and immunostaining with specific antibodies the remaining endogenous full-length A-RAF was found associated with vesicular structures. A-RAF positive vesicles were localized in the periplasmic area and at nearly each plus end of microtubules (Figure 3C). A significant fraction of A-RAF-positive punctate structures is lining the microtubular network that extends to the perinuclear area. A-RAF siRNA removed the punctate labeling, but not the diffuse staining over the nucleus that was also seen with normal rabbit IgG, (Fig S4C) and is therefore considered non-specific background. Consistently in cell fractionation experiments a significant part of A-RAF fractionates with cytoskeletal, but not with the nuclear fraction (Figure 3B).

### Overexpressed AR149 exhibits a dominant inhibitory effect on transport and traps internalized transferrin in ARF6 and RAB11 positive endosomes

**Figure 4:**
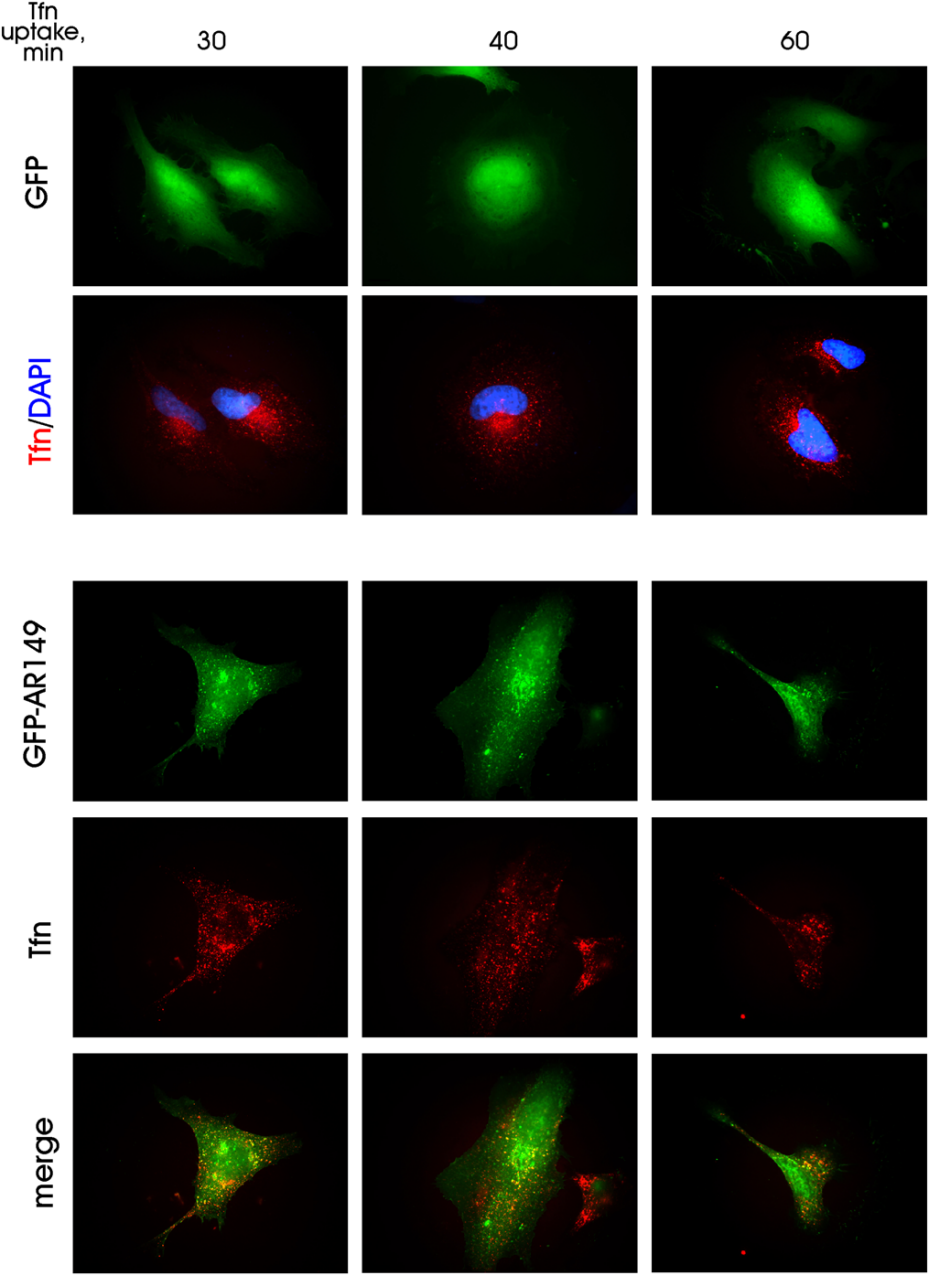
Expression of GFP-AR149 inhibits maturation of endosomes. GFP-AR149-transfected and control (GFP-transfected) cells were incubated with fluorescent Tfn for indicated times. Tfn containing vesicles accumulate in the pericentriolar area of control cells, but remain in the GFP-AR149 positive vesicles scattered throughout the cytosol of GFP-AR149 transfected cells. Representative images are shown.

**Figure 5:**
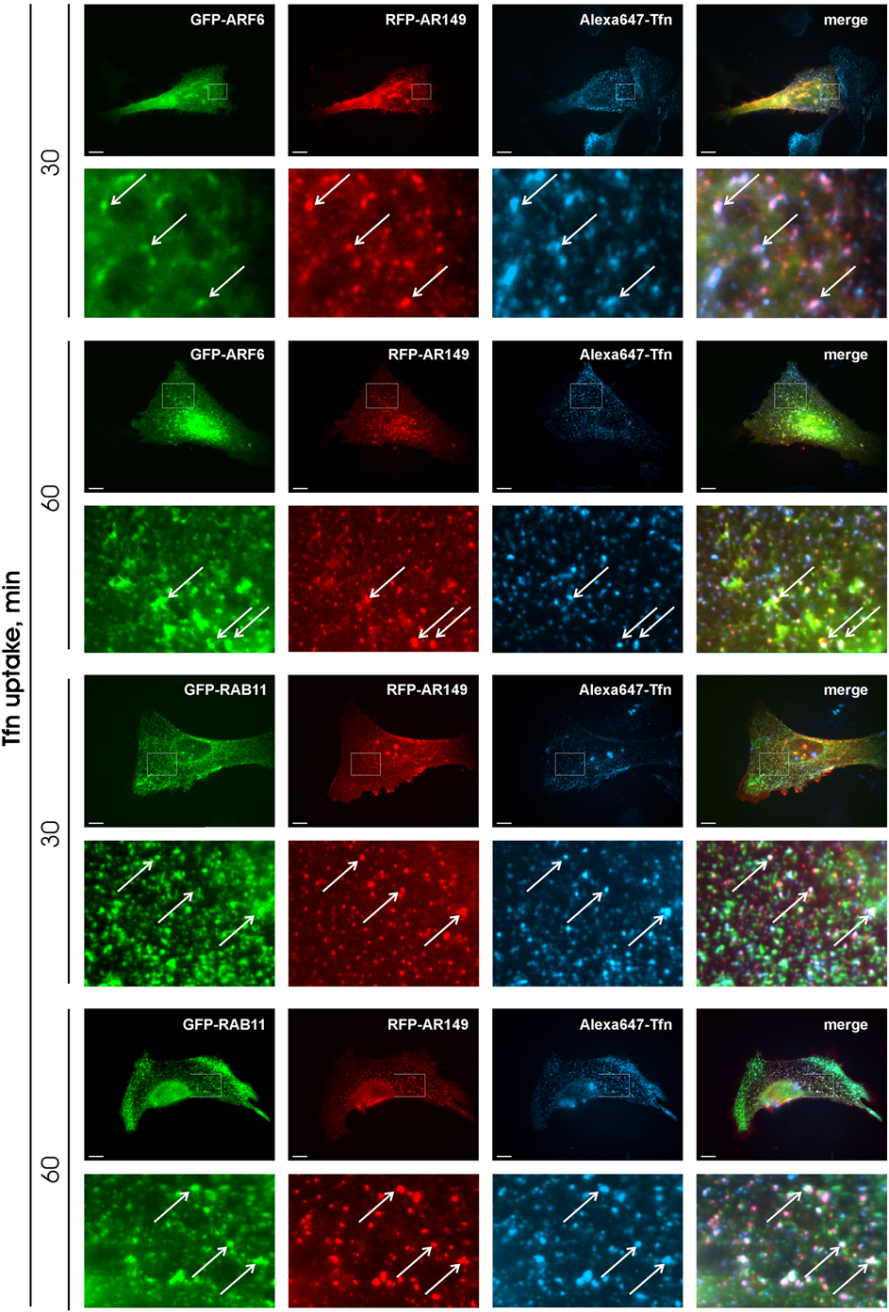
AR149 traps inernalized transferrin in ARF6 and RAB11 positive endosomes. HeLa cells were transfected with RFP-AR149 and either GFP-ARF6 or RAB11 as indicated. Uptake of fluorescent Tfn was examined after 30 and 60 minutes. Representative images show that Tfn is trapped by AR149 in GFP-ARF6 positive and in GFP-RAB11 positive vesicles. Enlarged areas are marked by boxes. Arrows indicate co-localization.

We asked whether overexpression of AR149 affects endocytic trafficking in mammalian cells. First, uptake and recycling of Tfn was investigated. Tfn is internalized upon binding to its receptor via a clathrin-dependent pathway and, after pH-regulated release, recycles back from the recycling/late endosomes. These endosomes are characterized by their accumulation in the pericentriolar space. GFP or GFP-AR149 transfected HeLa cells were incubated with Alexa-546 labelled Tfn for the indicated time (Figure 4). In control cells, after 30 to 40 minutes, endocytosed Tfn accumulated in the pericentriolar compartment (Figure 4, GFP panel). In GFP-AR149 expressing cells, relocation of endocytosed Tfn to pericentriolar compartments was significantly inhibited (Figure 4, GFP-AR149 panel). The difference is clearly visible at the 40 min timepoint, where transfected and non-transfected cells are next to each other. Therefore expression of AR149 does not influence the endocytic uptake of Tfn, but strongly inhibits its relocation to recycling endosomes. To pinpoint the cellular endosome compartment, which Tfn is trapped in, EEA1, RAB11 and ARF6 were used as markers for early and recycling endosomes. The data clearly show significant overlap between AR149, internalized Tfn and ARF6- as well as between AR149, Tfn and RAB11-positive vesicles (Figure 5). Consistently, EEA1 positive vesicles did not accumulate Tfn (Figure S5). Thus we conclude that the block in endocytic trafficking is at the level of tubular recycling endosomes.

### siRNA silencing of A-RAF and MEK inhibition mimicks the AR149 overexpression phenotype

The splice variant DA-RAF was shown to function as an effective inhibitor of RAF-ERK signaling [19]. As AR149 is expected to share this function, AR149 overexpression or A-RAF depletion should have the same effect on Tfn trafficking. For depletion we prepared a set of A-RAF specific siRNAs and transfected them into HeLa cells. Degree of depletion and specificity of A-RAF siRNAs were determined by immunoblotting. As documented in Figure S4A significant depletion of A-RAF but not of the other two RAF isoforms was achieved. Semi-quantitative RT PCR showed that siRNA down-regulates A-RAF mRNA selectively without affecting the mRNA levels of DA-RAF (Figure S4B), which was expressed at a lower level than full length A-RAF in this cell line.

**Figure 6:**
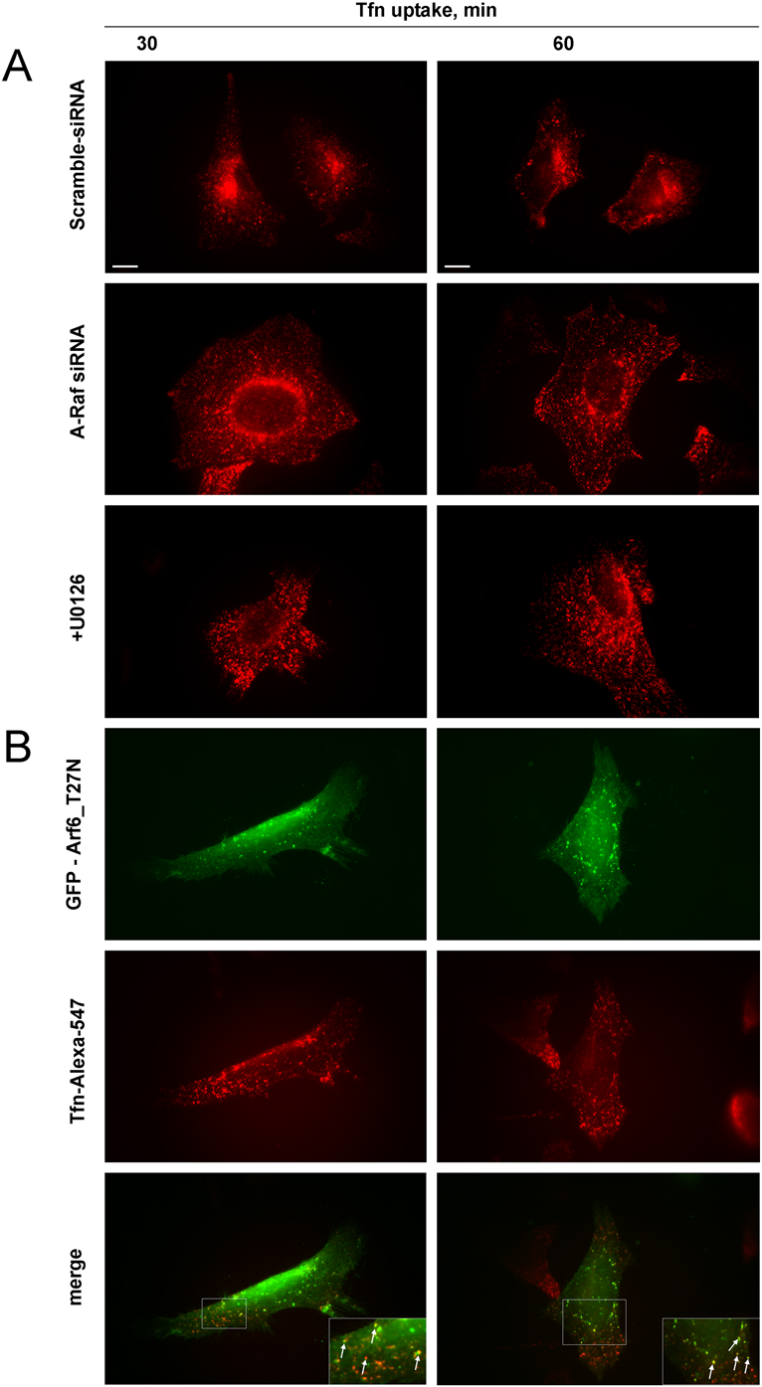
siRNA mediated A-RAF depletion, MEK inhibition and expression of ARF6(T27N) also block accumulation of Tfn in the pericentriolar endosome compartment. *A*. HeLa cells transfected with A-RAF siRNA, scrambled siRNA or treated with MEK inhibitor UO126 were subjected to Tfn uptake assay. Lack of accumulation of endocytosed Tfn in the pericentriolar compartment is similar to AR149-expressing cells (compare with Figure 4). Representative images are shown. Scale bar = 10 µm. B. HeLa cells were transfected with ARF6(T27N) before assay for fluorescent Tfn uptake. ARF6(T27N) positive vesicles accumulate internalised Tfn. The pattern of distribution is similar to that in A-RAF siRNA and to U0126 treated cells. Representative images are shown. Scale bar = 10 µm.

To test the effect of A-RAF depletion on Tfn trafficking, A-RAF siRNA-transfected, and control (scrambled siRNA-transfected) cells were subjected to a Tfn uptake assay. Similar to GFP-AR149 expressing cells, defective or significantly delayed accumulation of endocytosed Tfn in the pericentriolar space was observed (Figure 6A) indicating that A-RAF kinase activity and thus the activation of the mitogenic cascade is required for normal Tfn trafficking. Consistent with a requirement of localized ERK activation, chemical inhibition of the mitogenic cascade by U0126, a specific inhibitor of the RAF effector MEK, prevented the aggregation of Tfn positive endosomes in a similar way as A-RAF depletion (Figure 6A).

As the phenotypes of A-RAF depletion and mitogenic cascade inhibition overlap, we conclude that localized ERK signalling is required for endosomal maturation and AR149/DA-RAF functions as a dominant negative inhibitor of A-RAF on endosomes.

### Expression of GDP-locked ARF6(T27N) also blocks accumulation of Tfn in the pericentriolar endosomal compartment

Dominant negative ARF6 has been shown previously to interfere with internalisation of Tfn [15]. To test whether the pattern of Tfn accumulation by ARF6(T27N) and AR149 are comparable transfected HeLa cells were examined for co-localization of internalized Tfn and ARF6(T27N) (Figure 6B). Inspection of Figure 4 and Figure 6A reveals similar re-distribution of Tfn in HeLa cells in which A-RAF has been knocked down or inhibited (AR149 or U0126) and HeLa cells that express ARF6(T27N). This data suggests that ARF6 may be a target for regulation by A-RAF on endosomes.

### Tfn accumulates in ARF6 positive and RAB11 positive vesicles after A-RAF knock down

**Figure 7:**
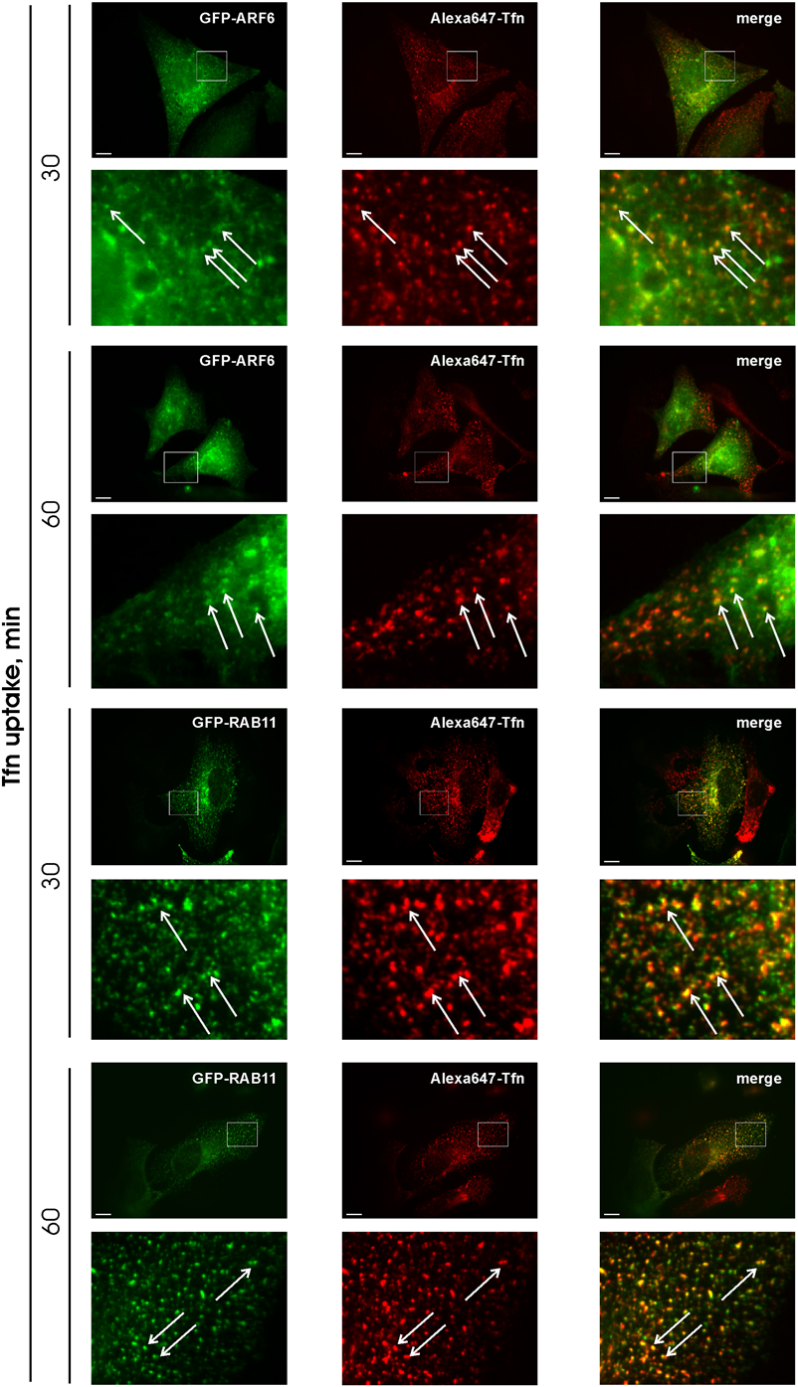
Tfn accumulates in ARF6 positives, RAB11 positives vesicles after A-RAF knock down. HeLa cells were cotransfected with either GFP-ARF6 or GFP-RAB11 and A-RAF siRNA. Tfn uptake was assayed as before. Tfn was found to co-localize with ARF6 or RAB11 as was observed in AR149 expressing cells. Enlarged areas are marked by boxes. Arrows indicate co-localization. Representative images are shown. Scale bar = 10 µm.

To characterize the endosome compartment in which Tfn accumulates after A-RAF knock down ARF6 and RAB11 were used as markers (Figure 7). Additionally we included EEA1 as marker of early endosomes (Figure S6). Co-localisation experiments in HeLa cells identify vesicles with accumulated Tfn as RAB11- and ARF6- positive. No EEA1 Tfn double positive vesicles were found. We conclude that the block in Tfn trafficking in the absence of A-RAF lies between tubular- and TGN-assotiated recycling endosome compartments.

### AR149 Interacts with ARF6 independent of nucleotide status

We have shown that AR149 and both (active and inactive) ARF6 mutants partially colocalize on endocytic vesicles and the plasma membrane (Figure S7). We were interested whether interaction of these two proteins could be shown at the biochemical level. HeLa cells were co-transfected with GFP, GFP-AR149 and HA-tagged ARF6 wild type, GTP-locked (Q67L) or GDP-locked (T27N) mutants. After immunoprecipitation with anti-GFP antibodies, the precipitates were separated by SDS PAGE and tested for bound ARF6 by immunoblotting against HA. In a reverse experiment ARF6 mutants were first precipitated and the presence of GFP-A-RAF in the precipitate was demonstrated by immunoblotting with anti-GFP antibodies.

**Figure 8:**
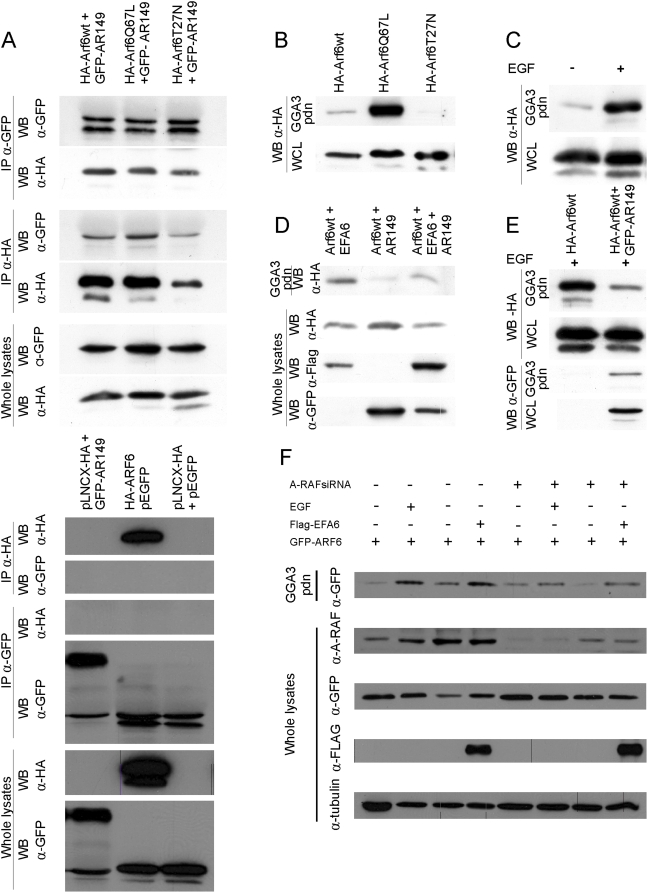
Down regulation of A-RAF by either AR149 or A-RAF siRNA interferes with activation of ARF6. A. Interaction of AR149 and ARF6. COS7 cells were co-transfected with GFP-AR149 and either HA-tagged ARF6wt, GTP-locked [ARF6(Q67L)] or GDP-locked [ARF6(T27N)]. After immunoprecipitation with α-GFP antibodies, co-precipitated ARF proteins were detected with α-HA antibodies. In the second experiment, precipitation and detection antibodies were exchanged. Expression levels in whole cell lysates (WCL) is shown in two bottom panels. Empty vectors were used for control (bottom panel). B. GGA3 interacts with ARF6•GTP. COS7 cells were transfected with wild type, GTP-locked (Q67L) or GDP-locked (T27N) HA-ARF6. Proteins pulled-down by incubation with GST-GGA3-Sepharose were detected with α-HA antibodies. C.,E. AR149 suppresses EGF-stimulated ARF6 activation. COS7 cells, transfected with either ARF6 alone or ARF6+GFP-AR149, were treated with EGF for 10 min and subjected to GGA3 pull-down. Bound protein was analysed by immunoblotting. Note decrease in the amount of ARF•GTP by AR149 coexpression. AR149 remains in the pulled-down ARF6 complex confirming the immunoprecipitation data. D. AR149 inhibits ARF6 activation by EFA6. COS7 cells were transfected with HA-ARF6 and either AR149, EFA6, or both. ARF6•GTP was pulled-down by GST-GGA3. AR149 decreases EFA6-stimulated ARF6 activation. F. ARF6 activation by EGF or EFA6 requires A-RAF. ARF6 activation was assessed by GGA3 pull-down. The amount of A-RAF and ARF6 protein were determed by Western blotting (WB). Treatment conditions are as indicated.

Figure 8A documents that similar amounts of ARF6, ARF6(Q67L) and ARF6(T27N) co-precipitated with GFP-AR149, irrespective of which protein was precipitated first. Thus interaction of A-RAF with ARF6 is independent of the ARF6 nucleotide status.

### A-RAF and AR149 regulate ARF6 activation

Next we addressed whether overexpression of dominant negative AR149 has an effect on activation of ARF6. ARF6 interacts specifically with its effector GGA3 in a nucleotide dependent manner. Therefore binding of GGA3 can be used as readout of the activation status of ARF6 [23]. In a control experiment, cell lysates from COS7 cells transfected with wild type, dominantly activated (Q67L) and dominant negative (T27N) mutants of HA-ARF6 were incubated with immobilized GST-GGA3 and washed. As shown in Figure 8B, GGA3 pulls effectively ARF6(Q67L), and less effectively ARF6wt. In contrast, no binding of ARF6(T27N) to GGA3 could be detected.

In subsequent experiments, COS7 cells were starved and induced with EGF, a strong elicitor of mitogenic signalling. Activated ARF6 was pulled-down by immobilized GGA3 as described above. Large amounts of ARF6 were pulled-down from EGF stimulated cells compared to non-stimulated cells (Figure 8C). Thus, ARF6 is activated by the mitogenic cascade. This activation is significantly diminished by the coexpression of AR149 (Figure 8E) or A-RAF knock down (Figure 8F).

Of note pulled-down ARF6 contains coexpressed AR149, which precludes competition of AR149 and GGA3 for binding to activated ARF6.

To pinpoint the position of AR149 action in a signalling pathway, COS7 cells were co-transfected with HA-ARF6 and either AR149, EFA6 or both. It has been previously described that overexpression of the ARF6 exchange factor, EFA6, increases the amount of ARF6 bound to GST-GGA3 beads. As depicted in Figure 8D, less ARF6 was pulled down in the presence of AR149. Similarly, A-RAF knock down reduced the amount of ARF6 that could be pulled-down with GST-GGA3. EFA6 partially rescued this effect (Figure 8F).

We conclude that AR149 co-expression or A-RAF knock down negatively affects the activation of ARF6 by EFA6, i.e. A-RAF functions upstream of ARF6.

## Discussion

### A-RAF is a distinct member of the RAF kinase family

In this work we unveil an unexpected link between A-RAF and regulation of ARF6 activity.

A-RAF possesses several features that set it apart from the other RAF kinases. *A-RAF* maps to the X chromosome and is the only steroid hormone-regulated *RAF* isoform, [32]. A-RAF protein has substitutions in a negatively charged region immediately upstream of the kinase domain (N-region), which is at least partially responsible for its low basal activity [12]. In contrast to C-RAF [33], [34], feedback phosphorylation of A-RAF by its downstream effector, ERK, has an activating effect on its kinase activity [35]. Interestingly, out of 590 kinases tested, the three RAF isoforms were among 208 kinases affecting clathrin- or caveolae-dependent endocytosis [36]. si-RNA mediated silencing of *A-RAF* inhibits, whereas that of *C*- and *B-RAF* activate SV40 uptake.

### Role of lipid binding domains, identification and properties of inhibitory A-RAF, AR149

Our initial studies on RAF distribution in a heterologous yeast system showed that of the three RAF isoforms, only A-RAF located to the cell cortex of yeast cells (Figure 1). This pattern resembles that of components of the endocytic machinery, such as Sla1p, Sla2p, clathrin light chain protein Clc1p [37]. Differences in localization of A-RAF and AR149 can be explained by the existence of two lipid-binding domains in the structure of A-RAF, each domain binding to specific sets of lipids.

The unique distribution pattern of A-RAF in yeast resembles the distribution of a PtdIns(4,5)P_2_ sensor, AP180 N-terminal homology (ANTH) domain [37]. Consistently, A-RAF is the only RAF isoform, which binds to immobilized PtdIns(4,5)P_2_ [27]. Using a set of point mutations, Johnson et al. (2005) located PtdIns(4,5)P_2_ binding site in the RAS binding domain of A-RAF. In contrast, we mapped PtdIns(4,5)P_2_ binding site to the cysteine rich domain of A-RAF because a RAS binding domain deleted mutant (88–606) still shows wild type distribution.

In agreement with Johnson et al (2005), replacement of arginine 52 in the RBD with leucine, which prevents binding of C-RAF RBD to RAS [38], disturbs the membrane localization of both full-length A-RAF and AR149 in yeast (Figure 1). We interpret this discrepancy by an interaction between RBD and CRD. It is known that RBD and CRD bind to RAS cooperatively [39]. The R52L mutation in the RBD may cause structural rearrangements disabling the interaction of the PtdIns(4,5)P_2_ binding site in the CRD with membranes.

CRDs are conserved among RAF isoforms, but only the A-RAF CRD possesses the unique property of PtdIns(4,5)P_2_ binding [27]. However as we show here the PtdIns(4,5)P_2_ binding site *per se* is not sufficient to specify the A-RAF wild type localization pattern. The wild type pattern of membrane binding requires in addition the PA binding site in the catalytic domain. In regard to the colocalization of A-RAF with ARF6 in mammalian cells, it is noteworthy that ARF6 is known to regulate the PtdIns(4,5)P_2_ content of membrane microdomains to which it binds.

In yeast Arf3p is the functional homolog of ARF6. Arf3p regulates PtdIns(4,5)P_2_ levels, endocytosis and actin polymerization [40], [41]. Therefore it is likely that the dominant lethal phenotype of AR149 in yeast is mediated by interference with Arf3p.

### Localization of A-RAF and AR149 in mammalian cells

The differences between RAF isoforms were most pronounced in yeast, where evidence for membrane attachment was restricted to A-RAF. The punctate cortical structures that are sites of A-RAF accumulation in yeast are known to contain proteins associated with early steps of endocytosis such as AP180 as well as Pan1p, that is associated with cortical actin patches. It is tempting to speculate that in mammalian cells the localization of A-RAF not only brings A-RAF into the neighbourhood of ARF6 on tubular endosomes, but additionally involves interaction with mammalian homologs of AP180/Pan1p that function at the interface of clathrin coated vesicles and an actin cytoskeleton regulatory complex. Such complexes are essential for endocytosis and also linked with the microtubule network [42]. Consistently GFP-AR149 decorated predominantly tubulo-vesicular endosomes, as confirmed by co-localization with ARF6 (Figure 3, middle panel). These endosomes lost their beads on the strings appearance upon Nocodazole treatment (Figure 3, top panel).

### Role of A-RAF and AR149 in endocytosis

Definitions of endocytic compartments are poorly standardized [43]. Here we used definitions given in [44], where endocytosed Tfn is first enclosed in small vesicles. These vesicles fuse to form early endosomes. Further maturation of early endosomes through tubular recycling endosomes leads to the pericentriolar TGN-associated recycling compartment. AR149 is targeted to tubular endosomes where it colocalizes with ARF6. Exogenous expression of AR149 or dominant negative ARF6(T27N) mutant causes trapping of endocytosed Tfn in tubular endosomes, which prevents transfer to pericentriolar recycling compartment and subsequent return to the plasma membrane. AR149 and ARF6 occur in the same complex as demonstrated both by fluorescence microscopy and coprecipitation experiments (Figure 8A).

A-RAF kinase was previously reported to participate in regulation of caveolae/raft-mediated endocytosis by stabilizing the caveolar coat [36], [45]. Our discovery of a requirement for A-RAF activity at a step subsequent to endocytosis, in the transfer to the recycling compartment, point to a broader role of A-RAF in membrane trafficking that additionally involves feedback regulation by the inhibitory splice variants DA-RAF 1,2 [19]. Our data suggest that DA-RAF does not necessarily work as a general inhibitor of mitogenic signalling as initially described [19]. More likely, due to its unique intracellular localization, AR149/DA-RAF primarily inhibits just a specific endosome-associated branch of mitogenic signalling, which is responsible for regulation of receptor recycling and/or restoration of signalling molecules.

The classical mechanism of cytoskeleton dependent endocytosis is subdivided in two parts: first, a short-distance step in the cortical area depends on actin and second long-distance microtubule dependent vesicular transport [42]. Our micrographs do not provide time resolution, but considering our functional analyses, it is conceivable that A-RAF functionally associates with the endosomes shuttling along microtubules between plasma membrane and the perinuclear recycling compartment.

From the functional analysis of Tfn endocytosis (Figure 6A), we conclude that not only localization to endosomes but also activity of A-RAF kinase in the mitogenic cascade on endosomes are a prerequisite for the translocation of Tfn-positive endosomes to the pericentriolar region.

### ARF6 regulation by A-RAF and AR149

Our data suggest that A-RAF functions upstream of ARF6. There has been an earlier report by Robertson [31], who suggested, based on epistasis experiments with ERK and ARF6 that ERK functions upstream and downstream of ARF6.

Several other reports also suggested dependence of ERK activation on ARF6 [17], [46], which is in direct contrast with the results of our epistasis analysis. This contradiction may be explained by a pleiotropic effect of active ARF6, which is known to block internalization of activated receptors [15], leading to sustained signalling through the mitogenic cascade. Our data additionally suggest EFA6 as a cooperation partner of A-RAF in the activation of ARF6. Of note inspection of the primary structure of EFA6 revealed multiple potential ERK/MAP kinase phosphorylation sites that suggest that EFA6 is a substrate of ERK downstream of A-RAF.

There are several examples for crosstalk between endocytosis and signal transduction [47], [48]. In addition, there is a growing body of evidence that at least part of mitogenic signalling takes place on endosomes: for example, the MEK partner MP1 was isolated as a component of endosomal vesicles and associates with MEK and participates in signal transduction [49]. Another scaffold of the mitogenic cascade, KSR1, was also shown to localize and mediate signalling to endosomes [31].

### Model of A-RAF function in regulation of endocytosis

**Figure 9:**
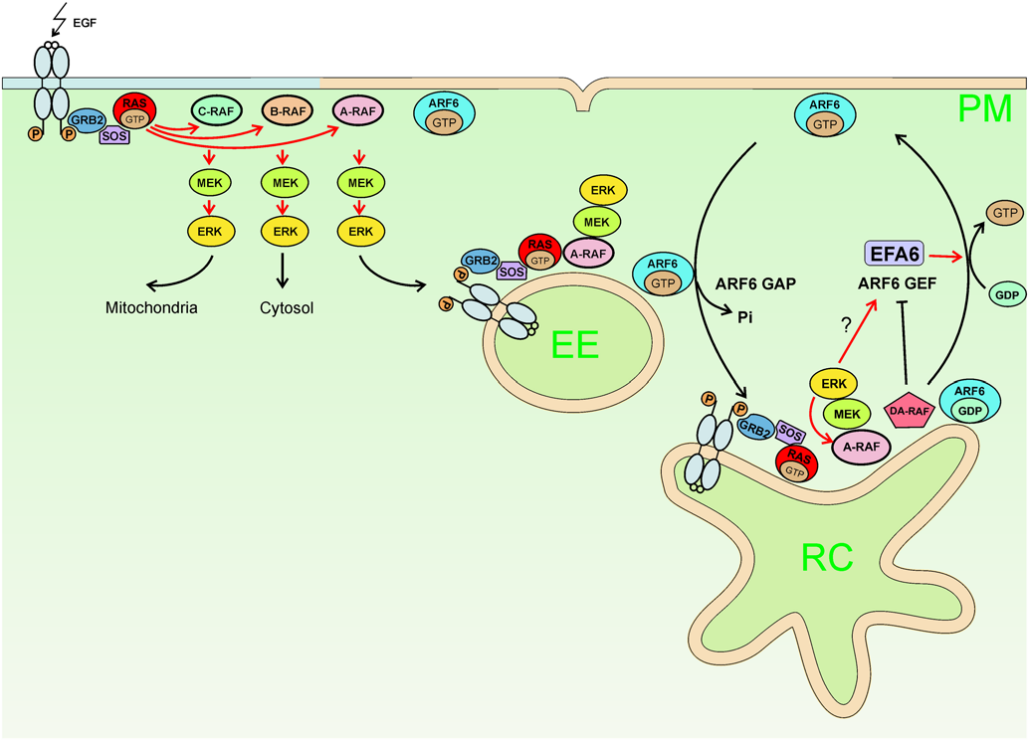
Model of A-RAF and AR149/DA-RAF function in regulation of endocytosis. Activation of receptor tyrosine kinase (here EGF receptor) leads to RAS-mediated activation of RAF kinases. RAF isoforms sort into different membrane microdomains, such as A-RAF into PtdIns(4,5)P_2_ rich domains. Activated ERK has opposing effects on A-RAF and C-RAF. Whereas A-RAF is activated, C-RAF becomes inactivated by feedback phosphorylation. A-RAF bound to PtdIns(4,5)P_2_ rich membranes continues to signal on endosomes leading to ARF6 activation. AR149/DA-RAF locates to recycling endosomes and blocks ERK activation in this compartment. See the main text for details. EE - early endosome, RC – recycling compartment, PM – plasma membrane. Red arrows indicate positive regulation of the process. Pale brown color indicates PtdIns(4,5)P_2_ rich membrane microdomains.

The novel findings on the A-RAF localization and the interaction with ARF6 have led to a new model of A-RAF function shown in Figure 9. Stimulation of growth factor receptors is followed by RAS activation. RAS•GTP recruits RAFs and leads to assembly of the RAF-MEK-ERK module at membranes. The three RAF isozymes become activated consecutively and mediate diversification of the signal to different subcellular compartments. A-RAF activation is delayed [50] because it has a corequirement for ERK phosphorylation [35], which uncouples A-RAF from B- and C-RAF that in turn are inhibited by feedback phosphorylation [33], [34]. Due to the unique localization of A-RAF to endosomes, this delay is optimal for regulation of later endocytic events such as recycling of receptors [51]. A-RAF function at endosomes also involves the mitogenic cascade and triggers ARF6 activation possibly via EFA6.

A-RAF involvement in ARF6 dependent endocytic recycling provides a new perspective for explaining the phenotype of A-RAF knock out mice, which exhibit severe neurological defects such as ataxia, rigidity of the musculature and continuous tremor [8]. Intriguingly, A-RAF, ARF6 and EFA6 are strongly expressed in Purkinje cells of mouse cerebellum [52], [53]. Along this line, DA-RAF expression is particularly strong in brain [19]. Endocytosis and rapid recycling of synaptic vesicles is critically important for the physiological function of neurons, which may further stress the role of A-RAF in the nervous system.

## Supporting Information

Table S1 List of primers used in this study.
(0.03 MB DOC)

Figure S1 A. Fractionation of yeast lysates by sucrose gradient centrifugation. Cell lysates were loaded on the top of sucrose gradient and centrifuged at 100.000×g. Fractionated lysates were loaded on SDS PAGE and immunoblotted. Proteins were visualized with specific antibodies. A-RAF is the only RAF protein, which segregates into heavy membrane/particle fractions. GFP alone fused with B- and C-RAF segregated into cytosolic/vacuolar fractions. Distribution of yeast membrane markers is shown in the lower rows. B. Immunoblot analysis of expressed GFP-A-RAF deletion mutants Yeast cell lysates expressing indicated GFP-A-RAF constructs were loaded on SDS PAGE and analyzed by Western blotting with antibodies against GFP. C. Lethality of GST-AR149. S.cerevisiae strain BJ 5459 was transformed with pEG-KT vehicle, pEG-A-RAF and pEG-AR149. Obtained colonies were streaked on uracildropout medium with glucose or galactose. Induction of protein production by galactose was lethal for GST-AR149 expressing cells, but not for those expressing either empty vehicle or full-length A-RAF. D. Part of GFP-A-RAF colocalizes with sites of endocytosis Yeast transformed with pUG36-AR149 and non-transformed control were incubated with lipophilic styryl dye FM 4–64 at 30°C for indicated time, washed and observed by flourescece microscopy. Some of the GFP-A-RAF positive spots overlap with sites of FM 4–64 uptake.
(5.70 MB TIF)

Figure S2 Mutational analysis of A-RAF with respect to its lipid binding properties. Basic residues in two presumptive lipid binding domains and in the RAS binding domain were replaced with leucine (R52) or alanine and subcellular distribution of mutant GFP fusion proteins was inspected by microscopy. Mutation of R359 and K360 in the Cterminal lipid binding domain (corresponding to phosphatidic acid binding domain of CRAF) gives the same distribution as deletion mutants which lost this domain. Mutation of R103 and K104 in CRD fully dislocated the protein into cytosol. R52L mutation, which is known to disturb the interaction of RAF with RAS had the same effect.
(3.38 MB TIF)

Figure S3 Effect of AR149 expression on the cytoskeleton of NIH 3T3 cells. NIH 3T3 cells were transfected with GFP-AR149 for 24 hours. After fixation, the polymerized actin was visualized with Alexa-546 conjugated phalloidin. Note the remarkable regression of actin stress fibers in the transfected cell. Scale bar = 10 µm.
(4.52 MB TIF)

Figure S4 Specific depletion of A-RAF protein and mRNA by siRNA A. HeLa cells were treated with A-RAF specific or scrambled siRNA and subjected to Western blot analysis with antibodies against actin (loading control) and three RAF isoforms. A-RAF is the only RAF isoform that decreased after siRNA treatment. B. HeLa cells were first treated with two different batches of A-RAF-specific or scrambled siRNA. Afterwards, the RNA was reverse transcribed and used as a template for quantitative PCR with primers specific for A-RAF, DA-RAF2 and Actin mRNAs. The ratio between the tested mRNA and actin mRNA was calculated from the qPCR data. From the diagram it can be concluded that A-RAF mRNA amount is decreasing significantly. DA-RAF2 mRNA was poorly expressed in these cells and its expression level did not change upon siRNA treatment. C. Controls of indirect immunofluorecent staining of endogenous A-RAF. HeLa cells were incubated with normal rabbit serum (left panel) or with A-RAF specific antibodies after A-RAF knock-down with siRNA (right panel). In both cases periplasmic punctate structures (see Fig. 3) disappeared, whereas nuclear staining remained. Scale bar = 10 µm.
(5.98 MB TIF)

Figure S5 Tfn does not accumulate in EEA1 positive early endosomes in AR149 expressing cells. HeLa cells were transfected as indicated and used for Tfn uptake assays. Note that fluorescence of Tfn and EEA1 do not mark identical vesicles. Enlarged areas are marked by boxes. Arrows indicate co-localization. Scale bar = 10 µm.
(9.23 MB TIF)

Figure S6 A-RAF knock down phenocopies the AR149 effect sparing EEA1 endosomes from Tfn accumulation. HeLa cells were transfected as indicated and used for Tfn uptake assays. Note that fluorescence of Tfn and EEA1 do not mark identical vesicles. Enlarged areas are marked by boxes. Arrows indicate co-localization. Scale bar = 10 µm.
(8.47 MB TIF)

Figure S7 AR149 colocalizes with dominant active and dominant negative ARF6 mutants in HeLa cells. RFP-AR149 was cotransfected with dominant active GFP-ARF6(Q67L) or dominant negative GFP-ARF6(T27N) and inspected by fluorescent microscopy. High degree of colocalization with both ARF6 mutants is documented in overlay figures. Scale bar = 10 µm.
(9.78 MB TIF)
