# Supplementary material for: A-RAF Kinase Functions in ARF6 Regulated Endocytic Membrane Traffic
Source: PLoS One. 2009 Feb 27;4(2):e4647. doi: 10.1371/journal.pone.0004647 (PMC2645234; doi:10.1371/journal.pone.0004647)
Supplement: Table S1 — List of primers used in this study. (0.03 MB DOC) [file pone.0004647.s001.doc]

**Supplemental Table Nekhoroshkova et al.**

List primers used in this work:

C-RAF and A-RAF deletion primers:

| A-RAF_for | 5’- CGC ACT AGT ATG GAG CCA CCA CGG GG-3’ |
| --- | --- |
| AR149_for | 5’- CGA GGA TCC ATG GAG CCA CCA CGG-3’ |
| AR149_rev | 5’- CGA AGC TTC TAG GTA GTG ATG TCA ACA CAG-3’ |
| AR149 (6*His) rev | 5’ – CGA AGC TTC TAA TGA TGA TGA TGA TGA TGG GTA CTC ATG TCA ACA CAG-3’ |
| A-RAF 1-230_for | 5’- CGA AGC TTC TAG TTG GAG TCC ATG GGG G-3’ |
| A-RAF 1-312_rev | 5’- GCG AAG CTT CTA CAG CTG CAC CTC ACT G-3’ |
| A-RAF 1-388_rev | 5’- CG AAG CTT CTA GCC CTC ACA CCA CTG TG-3’ |
| A-RAF 88-606_for | 5’-GCG ACT AGT ATG GTC GAG GTC CTT GAA GAT-3’ |
| A-RAF 142-606_for | 5’- GCG ACT AGT ATG ACA GTC TGT GTT GAC ATG AG-3’ |
| A-RAF 205-606_for | 5’- CTC CCG GGA TGG CCC CCC TAC AGC GCA TC-3’ |
| A-RAF 306-606_for | 5’- CTC CCG GGA TGC CAC CCA GTG AGG TGC AG-3’ |
| A-RAF 606_rev | 5’- CGA AGC TTC TAA GGC ACA AGG CGG G-3’ |
| A-RAF-RBD_for | 5’- CGC AAG CTT CAT ATG GTG GGC ACC GTC AAA G-3’ |
| A-RAF_RBD­_rev | 5’- GCG CTC GAG CTA CAG CGG GAC ATC TTC-3’ |

Mutagenic primers:

| A-RAF_ R52L_for | 5’- GAC AAG GCC CTG AAG GTG CTG GGT CTA AAT CAG GAC TGC-3’ |
| --- | --- |
| A-RAF_R52L_rev | 5’- GCA GTC CTG ATT TAG ACC CAG CAC CTT CAG GGC CTT GTC – 3’ |
| A-RAF_R103A, K104A_for | 5’- C ATG CAC AAT TTT GTA GCG GCG ACC TTC TTC AGC CTG-3’ |
| ARAF_R103A, K104A_rev | 5’- CAG GCT GAA GAA GGT CGC CGC TAC AAA ATT GTG CAT G-3’ |
| ARAF_R359A, K360A_for | 5’- GAG ATG CAG GTG CTC GCG GCG ACG CGA CAT GTC AAC-3’ |
| ARAF_R359A, K360A_rev | 5’- GTT GAC ATG TCG CGT CGC CGC GAG CAC CTG CAT CTC-3’ |
| ARF6(Q67L)_for | 5’- GGA TGT GGG CGG CCT AGA CAA GAT CCG GCC-3’ |
| ARF6(Q67L)_rev | 5’- GGC CGG ATC TTG TCT AGG CCG CCC ACA TCC-3’ |
| ARF6(T27N)_for | 5’- GGA CGC GGC CGG CAA GAA CAC AAT CCT GTA CAA G -3’ |
| ARF6(T27N)_rev | 5’- CTT GTA CAG GAT TGT GTT CTT GCC GGC CGC GTC C-3’ |
