# Supplementary figures and images for: A-RAF Kinase Functions in ARF6 Regulated Endocytic Membrane Traffic
Source: PLoS One. 2009 Feb 27;4(2):e4647. doi: 10.1371/journal.pone.0004647 (PMC2645234; doi:10.1371/journal.pone.0004647)

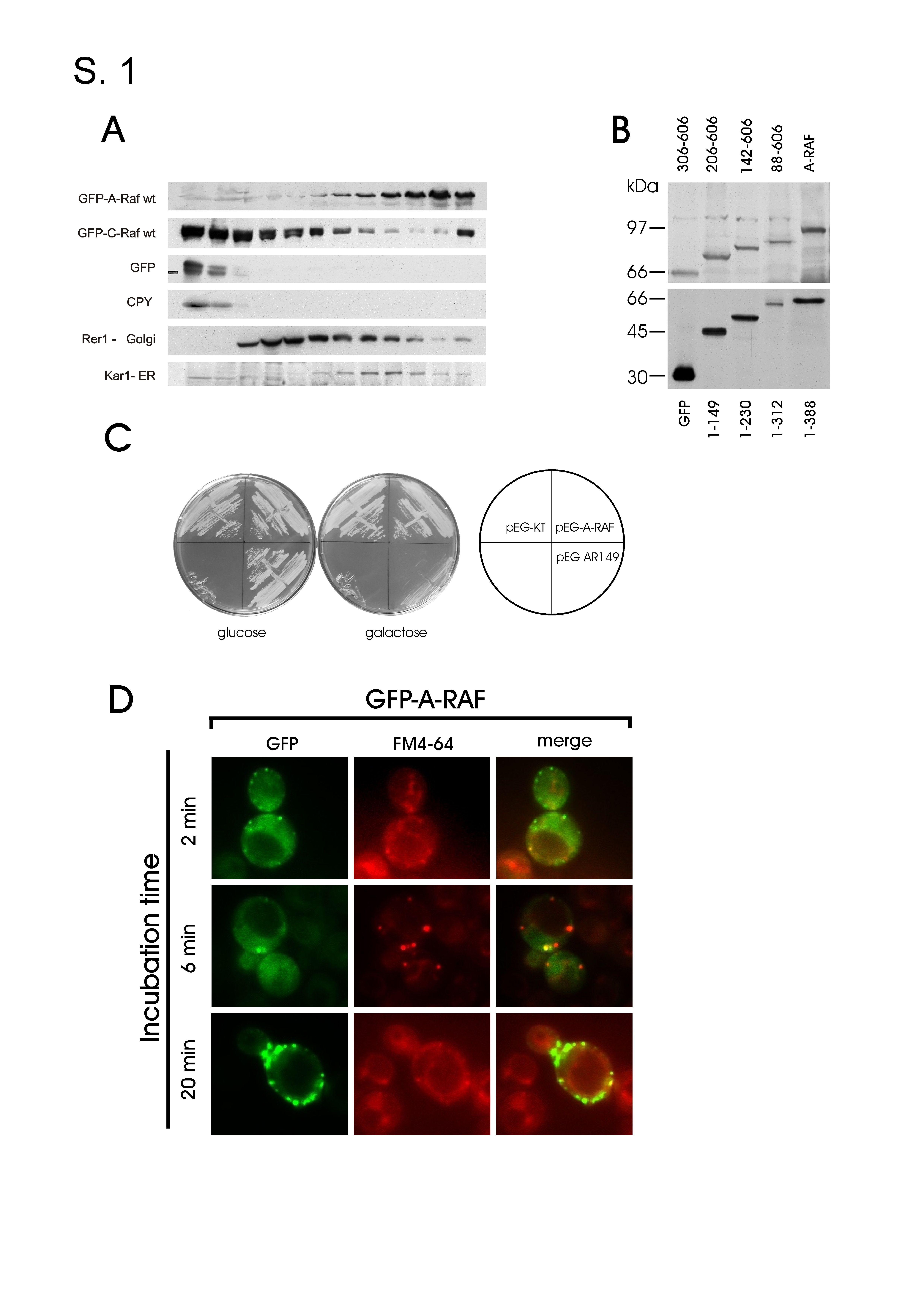

Supplement: Figure S1 — A. Fractionation of yeast lysates by sucrose gradient centrifugation. Cell lysates were loaded on the top of sucrose gradient and centrifuged at 100.000×g. Fractionated lysates were loaded on SDS PAGE and immunoblotted. Proteins were visualized with specific antibodies. A-RAF is the only RAF protein, which segregates into heavy membrane/particle fractions. GFP alone fused with B- and C-RAF segregated into cytosolic/vacuolar fractions. Distribution of yeast membrane markers is shown in the lower rows. B. Immunoblot analysis of expressed GFP-A-RAF deletion mutants Yeast cell lysates expressing indicated GFP-A-RAF constructs were loaded on SDS PAGE and analyzed by Western blotting with antibodies against GFP. C. Lethality of GST-AR149. S.cerevisiae strain BJ 5459 was transformed with pEG-KT vehicle, pEG-A-RAF and pEG-AR149. Obtained colonies were streaked on uracildropout medium with glucose or galactose. Induction of protein production by galactose was lethal for GST-AR149 expressing cells, but not for those expressing either empty vehicle or full-length A-RAF. D. Part of GFP-A-RAF colocalizes with sites of endocytosis Yeast transformed with pUG36-AR149 and non-transformed control were incubated with lipophilic styryl dye FM 4–64 at 30°C for indicated time, washed and observed by flourescece microscopy. Some of the GFP-A-RAF positive spots overlap with sites of FM 4–64 uptake. (5.70 MB TIF) [file pone.0004647.s002.tif]

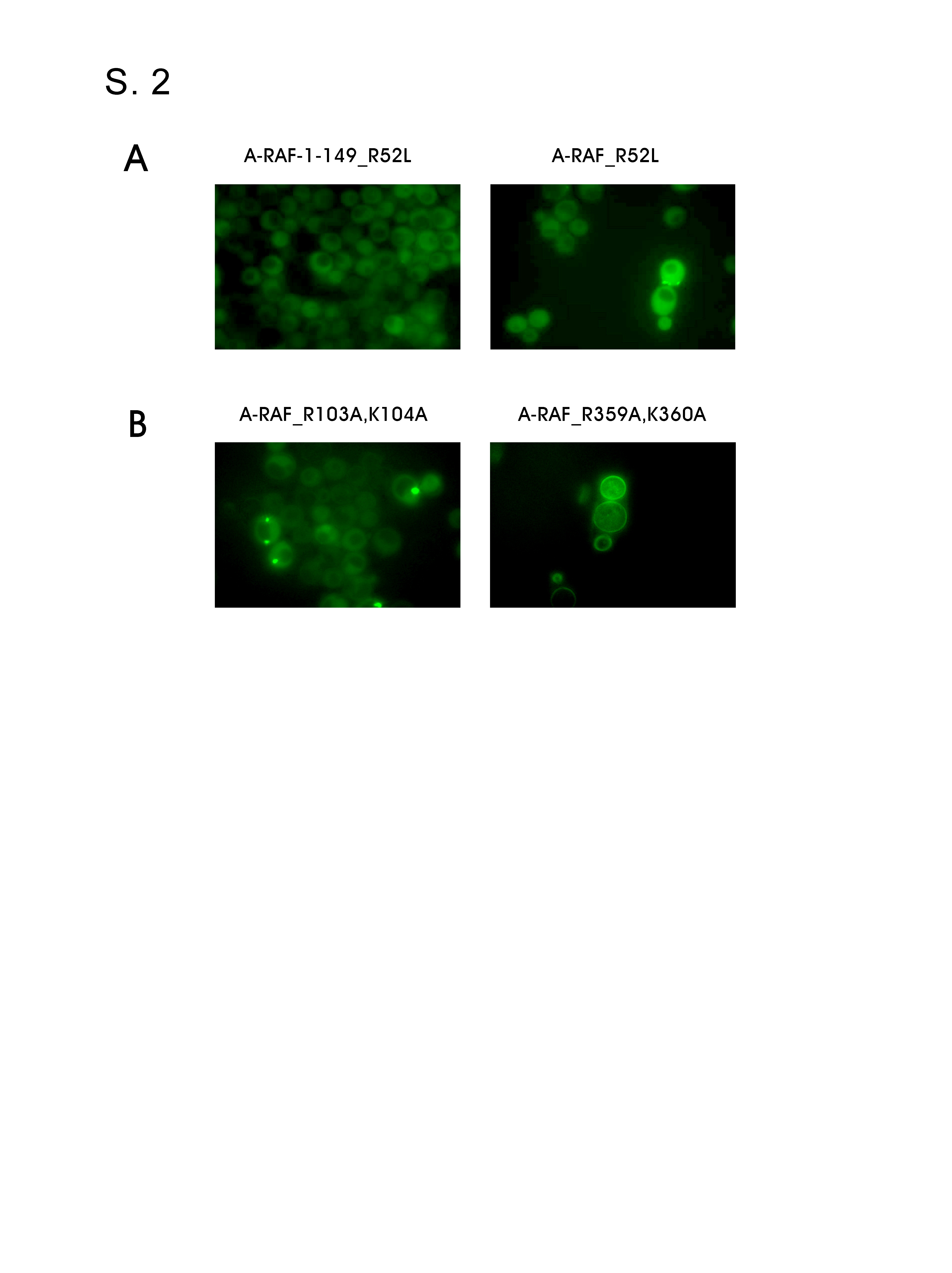

Supplement: Figure S2 — Mutational analysis of A-RAF with respect to its lipid binding properties. Basic residues in two presumptive lipid binding domains and in the RAS binding domain were replaced with leucine (R52) or alanine and subcellular distribution of mutant GFP fusion proteins was inspected by microscopy. Mutation of R359 and K360 in the Cterminal lipid binding domain (corresponding to phosphatidic acid binding domain of CRAF) gives the same distribution as deletion mutants which lost this domain. Mutation of R103 and K104 in CRD fully dislocated the protein into cytosol. R52L mutation, which is known to disturb the interaction of RAF with RAS had the same effect. (3.38 MB TIF) [file pone.0004647.s003.tif]

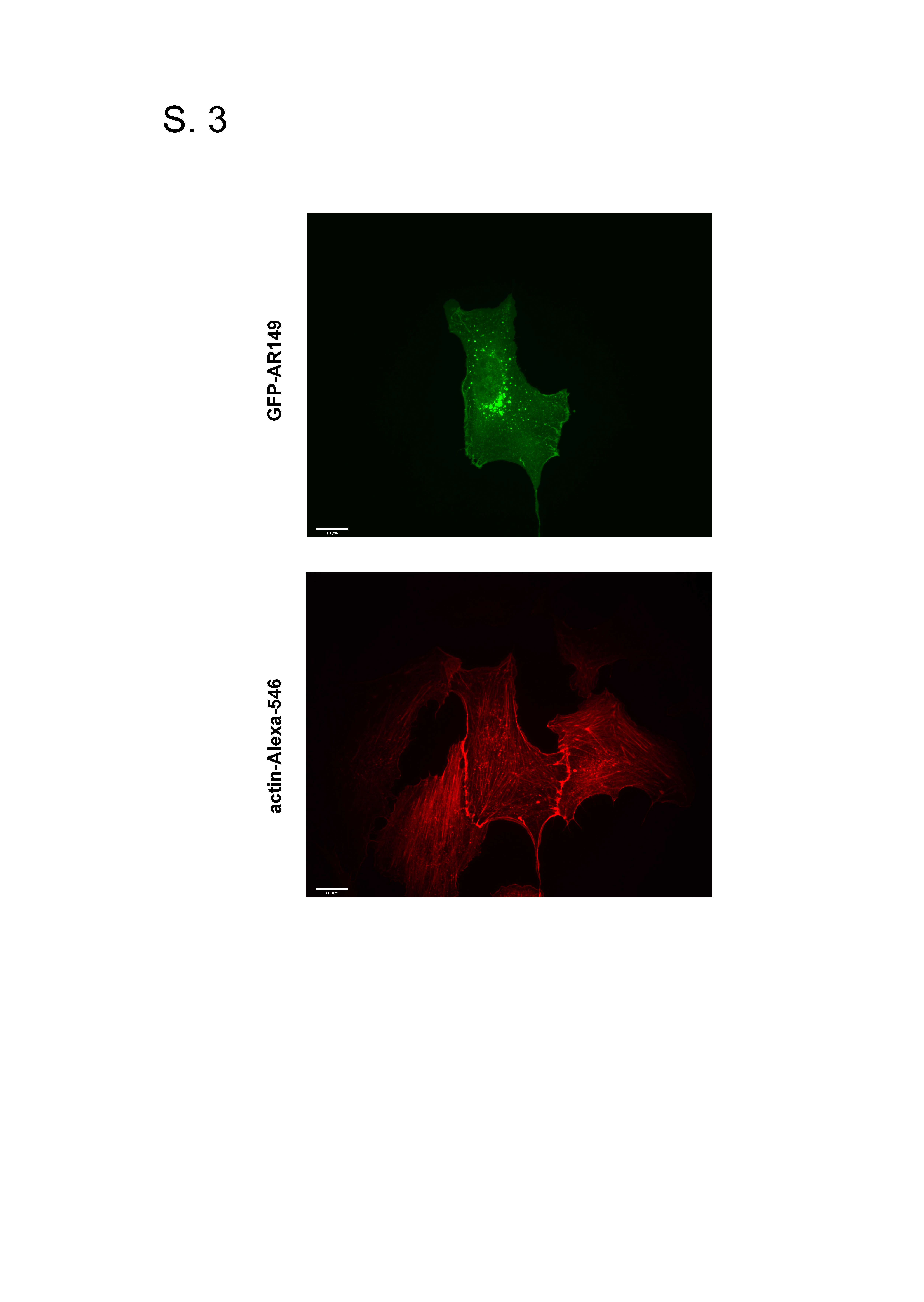

Supplement: Figure S3 — Effect of AR149 expression on the cytoskeleton of NIH 3T3 cells. NIH 3T3 cells were transfected with GFP-AR149 for 24 hours. After fixation, the polymerized actin was visualized with Alexa-546 conjugated phalloidin. Note the remarkable regression of actin stress fibers in the transfected cell. Scale bar = 10 µm. (4.52 MB TIF) [file pone.0004647.s004.tif]

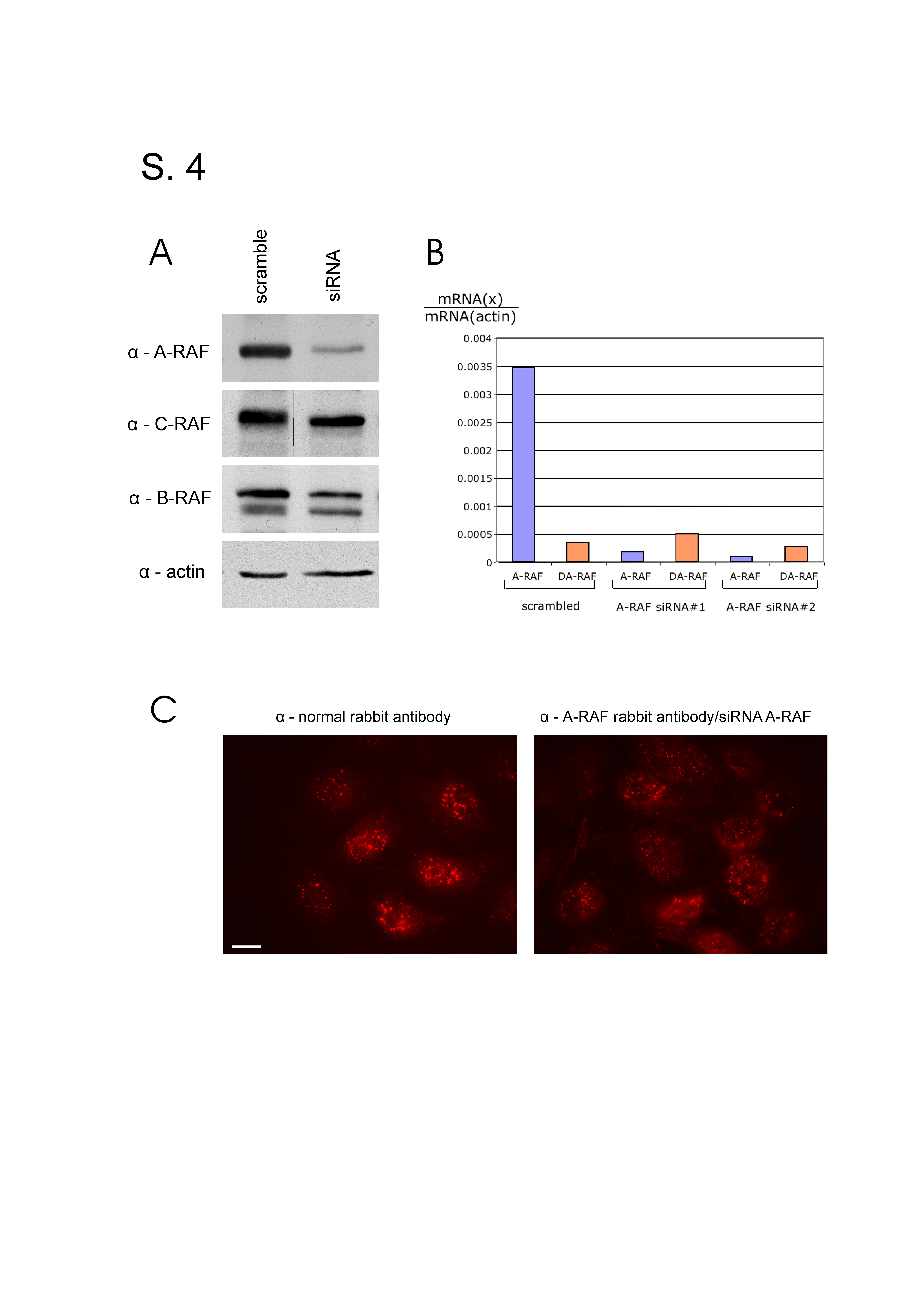

Supplement: Figure S4 — Specific depletion of A-RAF protein and mRNA by siRNA A. HeLa cells were treated with A-RAF specific or scrambled siRNA and subjected to Western blot analysis with antibodies against actin (loading control) and three RAF isoforms. A-RAF is the only RAF isoform that decreased after siRNA treatment. B. HeLa cells were first treated with two different batches of A-RAF-specific or scrambled siRNA. Afterwards, the RNA was reverse transcribed and used as a template for quantitative PCR with primers specific for A-RAF, DA-RAF2 and Actin mRNAs. The ratio between the tested mRNA and actin mRNA was calculated from the qPCR data. From the diagram it can be concluded that A-RAF mRNA amount is decreasing significantly. DA-RAF2 mRNA was poorly expressed in these cells and its expression level did not change upon siRNA treatment. C. Controls of indirect immunofluorecent staining of endogenous A-RAF. HeLa cells were incubated with normal rabbit serum (left panel) or with A-RAF specific antibodies after A-RAF knock-down with siRNA (right panel). In both cases periplasmic punctate structures (see Fig. 3) disappeared, whereas nuclear staining remained. Scale bar = 10 µm. (5.98 MB TIF) [file pone.0004647.s005.tif]

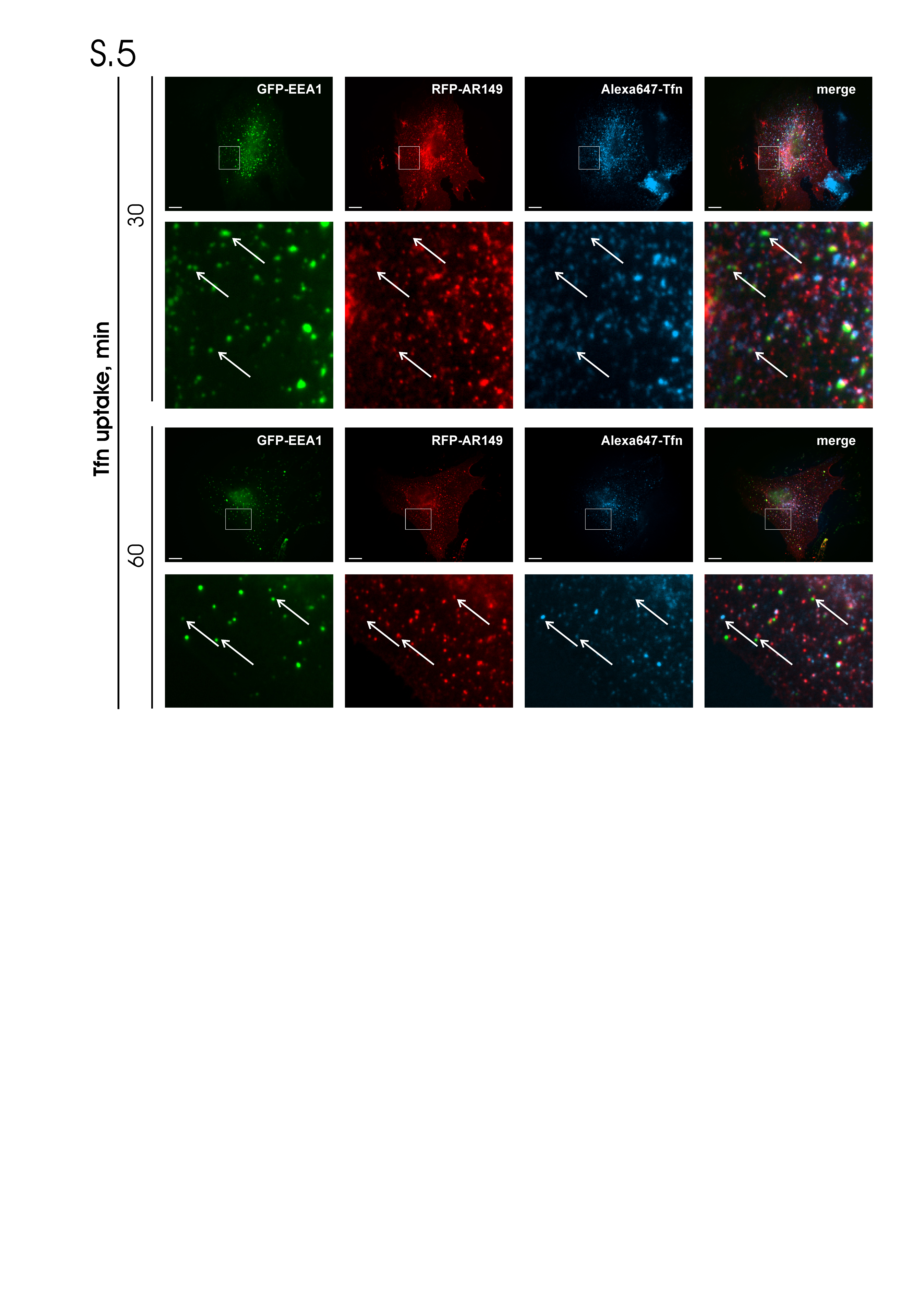

Supplement: Figure S5 — Tfn does not accumulate in EEA1 positive early endosomes in AR149 expressing cells. HeLa cells were transfected as indicated and used for Tfn uptake assays. Note that fluorescence of Tfn and EEA1 do not mark identical vesicles. Enlarged areas are marked by boxes. Arrows indicate co-localization. Scale bar = 10 µm. (9.23 MB TIF) [file pone.0004647.s006.tif]

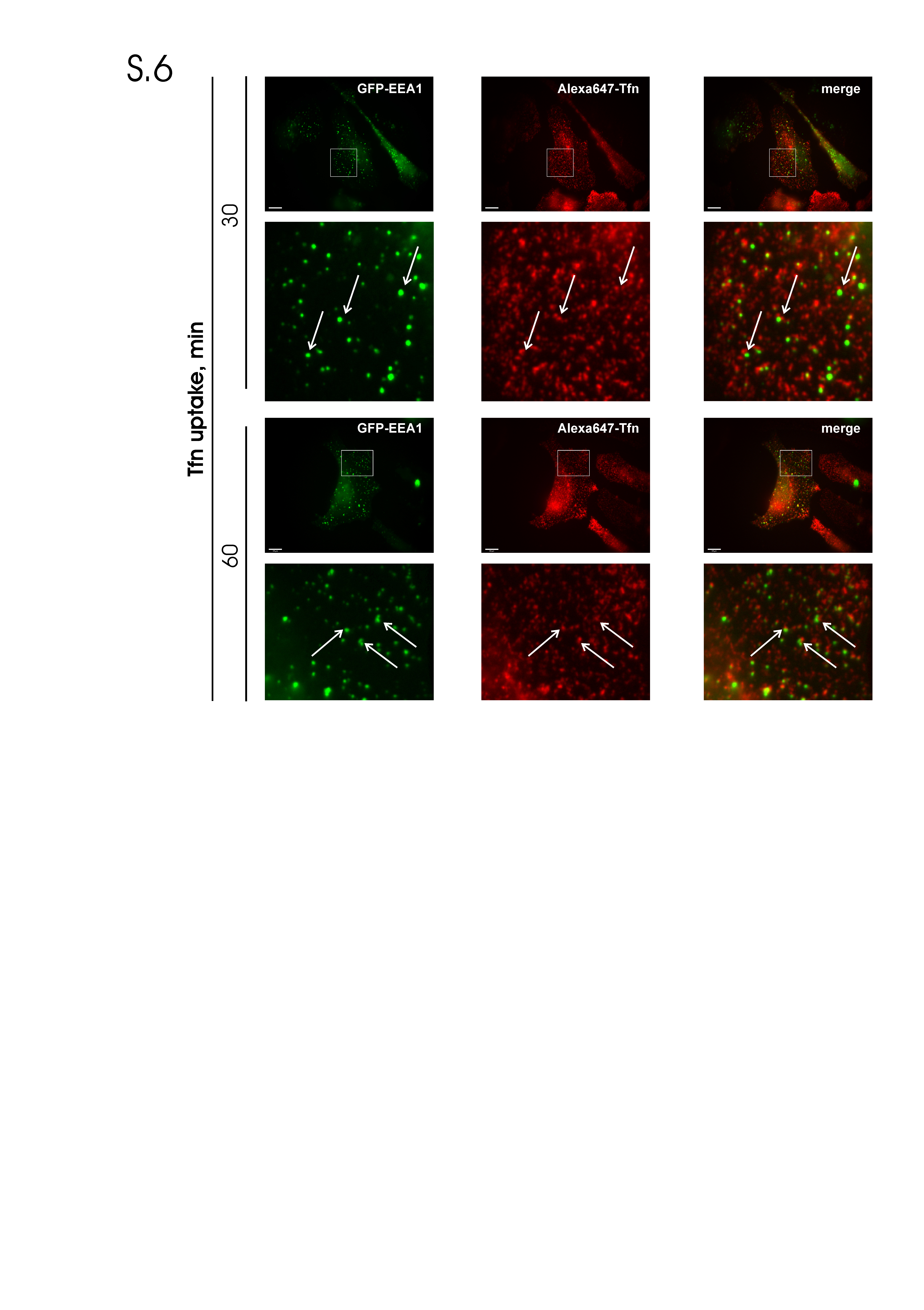

Supplement: Figure S6 — A-RAF knock down phenocopies the AR149 effect sparing EEA1 endosomes from Tfn accumulation. HeLa cells were transfected as indicated and used for Tfn uptake assays. Note that fluorescence of Tfn and EEA1 do not mark identical vesicles. Enlarged areas are marked by boxes. Arrows indicate co-localization. Scale bar = 10 µm. (8.47 MB TIF) [file pone.0004647.s007.tif]

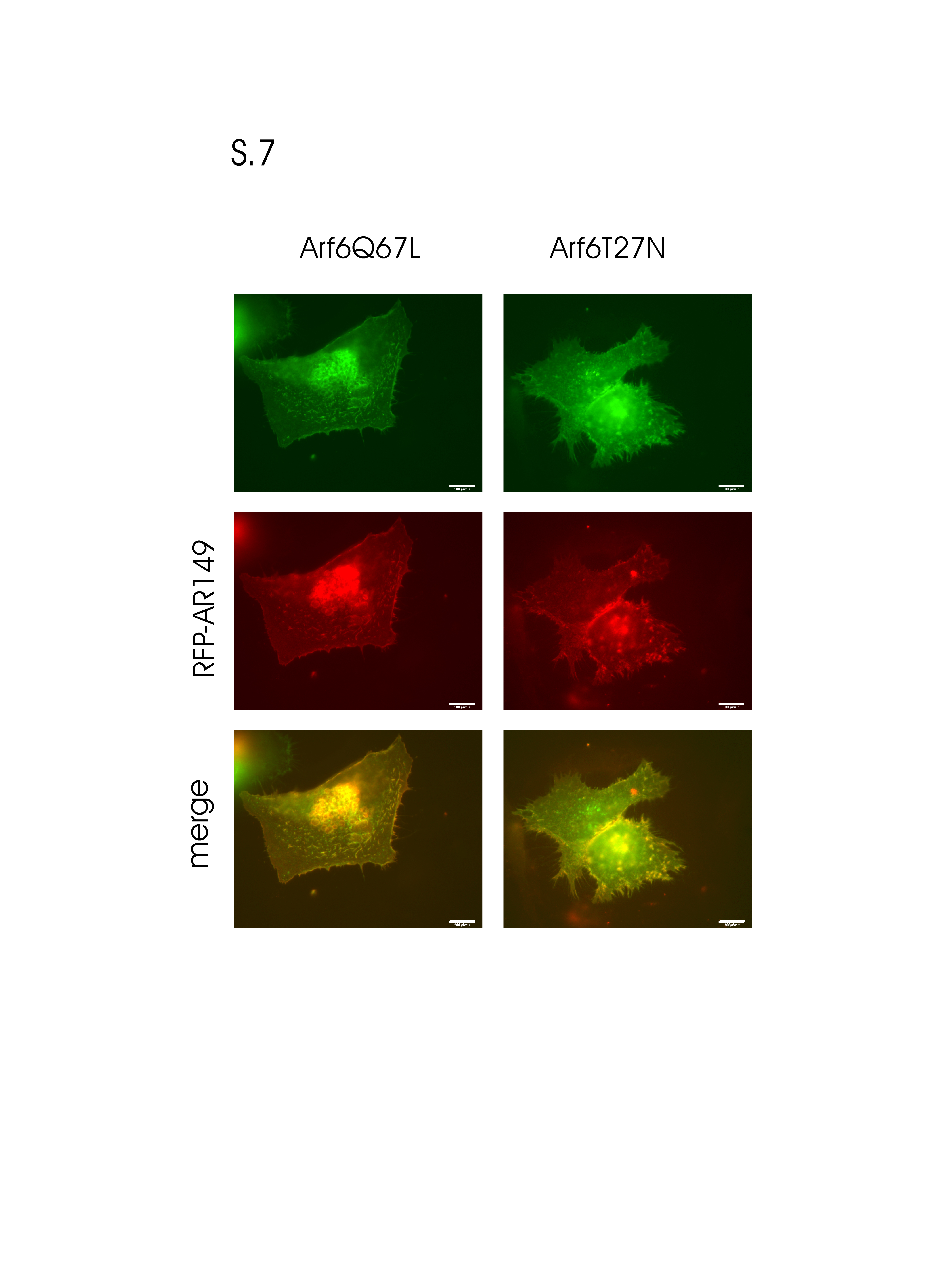

Supplement: Figure S7 — AR149 colocalizes with dominant active and dominant negative ARF6 mutants in HeLa cells. RFP-AR149 was cotransfected with dominant active GFP-ARF6(Q67L) or dominant negative GFP-ARF6(T27N) and inspected by fluorescent microscopy. High degree of colocalization with both ARF6 mutants is documented in overlay figures. Scale bar = 10 µm. (9.78 MB TIF) [file pone.0004647.s008.tif]
